# Tumor-infiltrating lymphocytes are functionally inactivated by CD90+ stromal cells and reactivated by combined Ibrutinib and Rapamycin in human pleural mesothelioma

**DOI:** 10.7150/thno.61209

**Published:** 2022-01-01

**Authors:** Haitang Yang, Sabina Berezowska, Patrick Dorn, Philipp Zens, Peiru Chen, Ren-Wang Peng, Thomas M. Marti, Gregor J Kocher, Ralph A. Schmid, Sean R.R. Hall

**Affiliations:** 1 Division of General Thoracic Surgery, Bern University Hospital, Bern, Switzerland.; 2 Department of BioMedical Research, University of Bern, Bern, Switzerland.; 3 Department of Thoracic Surgery, Shanghai Chest Hospital, Shanghai Jiao Tong University, Shanghai, People's Republic of China.; 4 Institute of Pathology, University of Bern, Bern, Switzerland.; 5 Graduate School for Health and Sciences, University of Bern, Switzerland.; 6 State Key Laboratory of Proteomics, Beijing Proteome Research Center, National Center for Protein Sciences, Beijing, People's Republic of China.

**Keywords:** malignant pleural mesothelioma, tumor microenvironment, tumor-infiltrating lymphocytes, exhaustion, reprogram

## Abstract

**Rationale:** Despite evidence suggesting that the tumor microenvironment (TME) in malignant pleural mesothelioma (MPM) is linked with poor prognosis, there is a lack of studies that functionally characterize stromal cells and tumor-infiltrating lymphocytes (TILs). Here, we aim to characterize the stromal subsets within MPM, investigate their relationship to TILs, and explore the potential therapeutic targets.

**Methods:** We curated a core set of genes defining stromal/immune signatures expressed by mesenchymal cells within the TME using molecular analysis of The Cancer Genome Atlas (TCGA) MPM cohort. Stromal and immune profiles were molecularly characterized using flow cytometry, immunohistochemistry, microarray, and functionally evaluated using T cell-activation/expansion, coculture assays and drug compounds treatment, based on samples from an independent MPM cohort.

**Results:** We found that a high extracellular matrix (ECM)/stromal gene signature, a high ECM score, or the ratio of ECM to an immune activation gene signature are significantly associated with poor survival in the MPM cohort in TCGA. Analysis of an independent MPM cohort (n = 12) revealed that CD8+ and CD4+ TILs were characterized by PD1 overexpression and concomitant downregulation in degranulation and CD127. This coincided with an increase in CD90+ cells that overexpressed PD-L1 and were enriched for ECM/stromal genes, activated PI3K-mTOR signaling and suppressed T cells. Protein array data demonstrated that MPM samples with high PD-L1 expression were most associated with activation of the mTOR pathway. Further, to reactivate functionally indolent TILs, we reprogrammed *ex vivo* TILs with Ibrutinib plus Rapamycin to block interleukin-2-inducible kinase (ITK) and mTOR pathways, respectively. The combination treatment shifted effector memory (T_EM_) CD8+ and CD4+ TILs towards T cells that re-expressed CD45RA (T_EMRA_) while concomitantly downregulating exhaustion markers. Gene expression analysis confirmed that Ibrutinib plus Rapamycin downregulated coinhibitory and T cell signature pathways while upregulating pathways involved in DNA damage and repair and immune cell adhesion and migration.

**Conclusions:** Our results suggest that targeting the TME may represent a novel strategy to redirect the fate of endogenous TILs with the goal of restoring anti-tumor immunity and control of tumor growth in MPM.

## Introduction

Malignant pleural mesothelioma (MPM) is a highly aggressive tumor affecting mesothelial cells lining the pleural cavity and is often associated with exposure to asbestos 1. The standard first-line treatment for MPM consists of platinum agents plus folate antimetabolites such as pemetrexed; however, the median survival time with chemotherapy remains between 12 and 18 months 2. Therefore, there is an urgent need to develop novel treatments for MPM.

A recent comprehensive integrative analysis of 74 cases of MPM identified diverse molecular features. Tumor suppressor genes defined subsets of MPM that were prognostic, despite a relatively low somatic mutational burden (TMB) (< 2 nonsynonymous mutations per megabase) and irrespective of histology 3. In addition, these molecular features were found to positively correlate with a 40-marker epithelial-to-mesenchymal transition (EMT)-related gene expression signature previously shown to be linked with poor prognosis in MPM 4. Importantly, the EMT score positively correlated with several genes known to negatively regulate the host immune system such as TGF-β1, TIM3, CTLA4 and PD-L2. TGF-β1 represented the top-ranked gene, which is a master regulator of EMT and cancer progression 5. In a large pan-cancer analysis, TGF-β signaling from cancer-associated fibroblasts (CAFs) positively correlated with a dysregulated extracellular matrix (ECM) transcriptional signature that was linked to immunosuppression and was a better predictor of failure to immune checkpoint blockade (ICB) targeting programmed death 1 (PD1) compared with other known biomarkers such as PD-L1 or TMB 6. Although studies have shown that CAFs exist in MPM 7, 8, little is known regarding their functions in the progression of MPM and resistance to treatment 8. Notably, CAFs represent a distinct cell type that has been identified using a small number of markers such as platelet-derived growth factor receptor alpha (PDGFRα) or fibroblast activation protein alpha (FAP), recent single-cell RNA sequencing data identified CAF subsets with unique functional properties in solid tumors of the breast 9, 10, pancreas 11 and lung 12. Whether this heterogeneity also translates in MPM is unknown.

MPM has generally been considered a nonimmunogenic tumor, which may relate to the low TMB and low levels of tumor-infiltrating lymphocytes (TILs) 13. Moreover, an immunosuppressive tumor microenvironment (TME) might also contribute to the ineffectual host immune response 14 and failure to respond to single-agent ICB 15. Despite evidence suggesting that the TME of MPM is linked with poor prognosis 16, there is a paucity of studies that functionally characterize mesenchymal cells and TILs within the TME.

In this study, we curated a core set of genes defining an ECM/stromal signature expressed by mesenchymal cells within the TME of various solid tumors 6, 17, 18 and explored their relationship to a curated immune activation (IA) CD8+ T effector cell signature 19 using molecular analysis of The Cancer Genome Atlas (TCGA) MPM cohort. We showed that a higher ECM/stromal to IA ratio is associated with shorter patient survival in MPM. In an independent MPM cohort, CD90+ cells overexpressing PD-L1 comprised a key source of ECM/stromal gene-related signature coinciding with PD1^hi^ CD8+ and CD4+ TILs with downregulated expression of the homeostatic regulator CD127. Despite this, TILs maintained their capacity to expand in a TCR-dependent manner *ex vivo* and ibrutinib plus rapamycin treatment was effective in reprogramming the TIL product to an effector memory TIL phenotype that re-expressed RA (CD45RA) (T_EMRA_) with diminished expression of exhaustion markers. Together, our findings suggest that combination therapy targeting the TME may represent a novel approach for TIL recovery leading to control of tumor growth in MPM.

## Methods

### TCGA database and establishment of ECM and IA gene signatures

Transcriptomic data of cancer patients were obtained from the TCGA (https://portal.gdc.cancer.gov/projects/TCGA). The gene expression and corresponding survival data were extracted for correlation and prognostic analysis using the corresponding packages in R (´corrplot´ and ´Hmisc´ packages for correlation analysis; 'maxstat', 'survival' and 'survminer' packages for prognostic analysis). ECM/stromal gene signature was scored as the sum of an ECM gene set (*VCAN, FAP, POSTN, FBLN1, COL1A1, PDPN, THY1, CSPG4, IL6, TGFB1, HGF, SERPINE1*) using genes that were shown to be highly overexpressed in CAFs and contribute to the EMT signature in various epithelial tumors 6, 17, 18. IA gene signature was scored as the sum of an immune gene set (*CD8A, GZMA, GZMB, GZMK, PRF1, IFNG, GNLY, IL2, CXCR3, IL7R, CD274, PDCD1, CTLA4*) 19, 20. Stratification of patients into high ECM (extracellular matrix/stromal) (in red) and low ECM (in black) or high IA (immune activation) (in red) and low IA (in black) gene signature scores is based on the optimal cutoff value of ECM gene signature score transcripts across all patients by using the surv_cutpoint function in R 'maxstat' package. For survival analysis, patients were grouped by gene expression, where 'high' and 'low' expression groups were stratified by the optimal cutoff value. Overall survival curves and cumulative hazard rates were analyzed and plotted by using R 'survival' and 'survminer' packages. The p-value was calculated using the log-rank test.

### Estimation of immune infiltration

To estimate the proportion of tumor-infiltrating lymphocytes (TILs) in solid tumors we used the computational method EPIC (Estimating the Proportions of Immune and Cancer cells), an algorithm that was specifically developed for RNA-sequencing data to estimate the proportions of different cell types from bulk gene expression data 21, 22. Briefly, normalized expression data (as transcripts per millions (TPM)) of TCGA MPM tumors were used as inputs and then quantified via deconvolution the proportions of immune cell types (CD8+ T and CD4+ T cells) using the “EPIC” package in R (https://github.com/GfellerLab/EPIC). Three algorithms (EPIC, MCPCOUNTER and TIDE) that estimate CAF signature score were also used to investigate the correlation between the gene expression of THY1/CD90 and CAFs 21.

### Immune subtype signatures

C1-6 immune subtype models were generated according to a previously curated dataset 23, 24. Briefly, C1 (wound healing) subtype had elevated expression of angiogenic genes and a high proliferation rate; C2 (IFN-g dominant) subtype had the highest M1/M2 macrophage polarization, a strong CD8 signal and, together with C6, the greatest TCR diversity, and also showed a high proliferation rate; C3 (inflammatory) subtype was characterized by elevated Th17 and Th1 genes and low to moderate tumor cell proliferation; C4 (lymphocyte depleted) subtype displayed a more prominent macrophage signature, with Th1 suppressed and a high M2 response; C5 (immunologically quiet) subtype exhibited the lowest lymphocyte and highest macrophage responses, dominated by M2 macrophages. The C6 (TGF-b dominant) subtype displayed the highest TGF-b signature and a high lymphocytic infiltrate with an even distribution of the type I and type II T cells.

### TCGA Reverse phase protein array dataset

We examined the correlative analysis (spearman's R) between phospho-mTOR (S2448), a biomarker indicating the activation of mTOR signaling and THY1/CD90 gene expression across TCGA MPM tumor samples. Protein quantification of phospho-mTOR (S2448) was based on Reverse phase protein array (RPPA) dataset from TCPA portal. Data were downloaded and reanalyzed from the TCGA MPM RPPA (reversed-phase protein array) dataset (http://tcpaportal.org/tcpa/) 25, 26. Normalized level 3 data were used, and the data normalization is processed as follows: 1). Calculate the median for each protein across all the samples; 2). Subtract the median (from step 1) from values within each protein; 3). Calculate the median for each sample across all proteins; 4). Subtract the median (from step 3) from values within each sample.

### Tissue procurement, generation of single cells and flow cytometry

MPM samples were collected at the University Hospital of Bern (see Table S1). All patients gave informed written consent for the usage of surgical material for research purposes, which was approved by the Ethics Commission of the Canton of Bern (KEK-BE: 042/2015). Fresh MPM tissue was dissociated to single cell suspension and quantification and prospective isolation of mesenchymal cell subsets, as well as analysis of TILs was performed as previously described 27. Following Fc block (eBioscience), single cells were incubated with a panel of fluorescently conjugated human monoclonal antibodies directed at the following epitopes: CD45, CD14, CD31, CD235a, CD73, CD90, EpCAM, PD-L1 (see Table S2). Cell acquisition was performed using a BD FACS LSRII SORP (BD Biosciences). A minimum of 1 x 10^5^ events was collected and analyzed using FlowJo ver10.7 (Tree Star). For cell sorting, CD90+ cells (see Figure S4 for full gating strategy) were sorted directly into collection buffer containing 20% FBS using a BD FACS Aria III or BD FACS Aria and expanded as previously described 28, 29. Following expansion, a portion of CD90+ cells were lysed with RLT buffer (Qiagen) and stored at -80 °C for RNA isolation at a later time point.

For analysis of TILs, single cell digests from the same patients were resuspended in 50µl of staining buffer (BD Biosciences) following Fc block (eBioscience) and stained with a panel of fluorescent antibodies against CD45, CD3, CD8, CD4, CD45RO, PD1, CD107a, CD127 (see Figure S2 for full gating strategy and Table S2 for details on antibodies). To discriminate live from dead cells, the cell dye 7-AAD (eBioscience) was added prior to analysis. Cell acquisition was performed using a BD FACS LSRII SORP (BD Biosciences). A minimum of 1 x 10^5^ events was collected and analyzed using FlowJo ver10.7 (Tree Star).

### Immunohistochemistry

Serial sections (2.5 μm) from formalin-fixed paraffin-embedded MPM samples taken before and after neoadjuvant chemotherapy were stained for anti-human PD-L1 (clone E1L3N, Cell Signaling Technology, Danvers, MA, USA) at a 1:400 dilution at room temperature for 15 min, followed by incubation with the secondary antibody using the Bond Polymer Refine Kit with 3-3'-Diaminobenzidine-DAB as chromogen (Leica Biosystems), counterstained with hematoxylin and mounted in Aquatex (Merck, Darmstadt, Germany). Tumoral PD-L1 expression was scored by a trained pathologist (SB) according to current guidelines for lung tumors as the percentage of cells with membranous staining of any intensity. The tumor proportion score based on percent (%) positivity consisted of the following groups: < 1; between 1 to < 50; and ≥ 50, as previously described (4). Additional sections were stained with anti-CD4 (clone 4B12, Novocastra) at 1:100 dilution and anti-CD8 (Dako, clone C8/144B) at a 1:100 dilution following antigen retrieval in Tris buffer solution at 95 °C for 20 min. Secondary antibody staining was carried out as described above. We assessed the density of CD8 positive TIL in the tumor area (positive cells/mm^2^) using a semi-automated approach with the QuPath software, version 0.1.2 30.

### Evaluation of CD90, αSMA and FAP co-expression in MPM

We evaluated the co-expression of CD90/aSMA and CD90/FAP on sequential sections of three patients represented all three histological subtypes in this cohort (BE09, biphasic; BE013, sarcomatoid; BE017, epithelioid). All samples included a pre-neoadjuvant chemotherapy (NAC) as well as the post-NAC were included. All sections were stained using the automated system BOND RX® (Leica Biosystems, Newcastle, UK), as previously described 31. Briefly, sections were stained with THY1/CD90 antibody (ab92574, Abcam) at a 1:200 dilution following antibody retrieval by heating slides in citrate buffer for 20 min at 95 °C: αSMA (1:60000, A-2547, Sigma) according to the manufacturer's instructions and FAP (1:200, ab207178, Abcam) following antibody retrieval by heating slides in Tris buffer for 30 min at 95 °C. Antibody detection was carried out using chromogenic staining procedures inside the staining system BOND RX® (Leica Biosystems). All sections were scanned at 40x using the Pannoramic P250 (3DHistech, Budapest, Hungary) resulting in a resolution of 0.1215 μm/px. The sections were aligned using QuPath 0.2.3 applying the affine method and three tiles of 5000 x 5000 pixels per section were annotated for manual evaluation of the co-expression: complete, all CD90 positive cells express the other marker; partial, some CD90 positive cells do not express the other marker; none, no co-expression.

### Flow cytometric analysis of expanded CD90+ cells

FACS sorted and expanded CD90+ cells were harvested and re-suspended in FACS staining buffer. Following Fc block, cells were incubated with the following fluorescently conjugated human monoclonal antibodies used to detect mesenchymal lineages: PDGFRα, PDGFRβ, NG2, TLR2, TLR3, TLR4, TLR9 (see Table S2). We performed antibody dilutions prior for each to carrying out the final immunophenotypic analysis. Cells were incubated on ice in the dark for 30 min. To exclude dead cells and debris, 7-AAD was added. Cell acquisition was performed using a BD FACS LSRII. For analysis, a minimum of 30,000 events was collected and analyzed using FlowJo software version 10.7 (Tree Star).

### RNA extraction and RT-qPCR

Total RNA was extracted from primary MPM-derived CD90+ cells using RNeasy Mini Kit (Qiagen) to analyze gene expression using real time quantitative PCR (RT-qPCR). Briefly, cDNA was synthesized using GoScript reverse-transcription system (Promega). RT-qPCR was performed in triplicates with target-specific primers using TaqMan Gene Expression Assay (Applied Biosystems) or dye-based detection with GoTaq PCR master mix (Promega) on AB7500 FAST real-time PCR system (Applied Biosystems). Expression levels were normalized to internal controls tested for expression stability across samples in each experiment using Expression Suite Software (Life Technologies). Relative expression was calculated by 2-ΔΔCT method. (See supplemental Table S3). For relative gene expression, RNA was extracted from the mesothelial cell line MeT-5A (ATCC^®^ CRL-944™) and set to one.

### Measurement of Annexin V and Propidium Iodide

Early passage CD90+ cells and various MPM cell lines (Meso1, Meso4, H28, H2052) were plated at 10 x 10^4^ cells per well in 6 well plates in their respective media. After 24 hs, media was replaced with serum-free media and cells were serum-starved for 24 h. Serum starved cells were treated with vehicle or 2.5 µM cisplatin with 5 µM pemetrexed or 5 µM cisplatin with 10 µM pemetrexed. Complete media changes with the new drug were added each day. After 72 h, cells were stained with Annexin V/PI staining kit (Invitrogen) according to the manufacturer's instructions. A minimum of 50,000 events was collected using a BD Bioscience LSR II (BD Biosciences). FCS files were analyzed using FlowJo ver 10.7 (TreeStar). An apoptotic score was calculated by summing the % of Annexin^+^PI^+^ and Annexin^+^PI^-^ subsets.

### T cell-activation assay and coculture with CD90+ cells from MPM

CD3^+^ T cells were isolated from healthy peripheral blood mononuclear cells (PBMCs) using the EasySep™ Human CD3 T Cell Isolation Kit (StemCell Technologies) according to the manufacturer's instructions and labeled with 0.5 µM carboxyfluorescein succinimidyl ester (CFSE) (eBioscience). CFSE-labeled CD3+ T cells (2 x 10^5^) were stimulated with 500 ng/ml Staphylococcal Enterotoxin B (Sigma-Aldrich) in 96-wells U-bottom plates in T cell media (Immunocult XF, StemCell Technologies) supplemented with 10 ng/ml of recombinant human IL-2 (Peprotech) at 37 °C and 5% CO_2_. Separately, FACS expanded CD90^+^ cells were gamma-irradiated (10 Gy) and treated with 50 ng/ml of rhTNFα (Gibco) and 50 ng/ml of rhIFNγ (Gibco) or vehicle for 24 h. 4 x 10^4^ or 2 x 10^5^ immune primed CD90+ MPCs were added to wells containing 2 x 10^5^ CFSE-labeled T cells following stimulation with SEB, as described above. In separate experiments, CD90+ MPCs were treated with 10 µg of human PD-L1 neutralizing antibody (BPS Bioscience) and 2.5 µg of human TGF-β1 neutralizing antibody (MAB240, clone 9016, R&D Systems) for 3 h in combination prior to co-culturing with SEB-activated CD3+ T cells. After 5 days of culture at 37 °C and 5% CO2, wells were harvested and cells were stained with anti-human CD45, anti-human CD4, anti-human CD8, anti-human PD1 and anti-human CD127 cocktail. Cytotoxic degranulation was detected by the addition of anti-human CD107a (eBioscience) and cell viability using 7-AAD (eBioscience) according to the manufacturer's protocol. To measure intracellular levels of IFNγ, cells were stained with the LIVE/DEAD™ Fixable Near-IR Dead Cell staining kit (Invitrogen), according to the manufacturer's instructions. Afterwards, cells were incubated with Fc block (Invitrogen) and in 100 µl of staining buffer with fluorochrome-conjugated CD107a-PE-TR. Cells were washed and resuspended in 100 of Cytofix/Cytoperm™ solution for 20 min at 4 °C. Cells were washed twice in 1X Perm/Wash™ solution. Afterwards, fixed/permeabilized cells were resuspended in 50 µl of Perm/Wash™ solution with anti-human IFNγ-APC antibody or appropriate isotype control and incubated at 4 °C for 30 min in the dark. After staining, cells were washed twice with 1X Perm/Wash™ solution and resuspended in PBS prior to flow cytometric analysis. A minimum of 50,000 events were collected using an LSR II BD Biosciences. FCS files were analyzed using FlowJo 10.7 (TreeStar).

### Expansion of TILs from MPM tissue

To expand naturally occurring, autologous TILs from bulk MPM tumor, following digestion of fresh MPM tissue, single cells were seeded in ultra-low adherent Nunclon sphere plates (Thermo Scientific) at 50 x 10^4^ cells/well in ImmunoCult™-XF T-cell media supplemented with rhIL-2 (10 ng/ml), rhIL-7 (10 ng/ml) and rhIL-15 (15 ng/ml) with 3% human AB serum. For T cell receptor (TCR) stimulation, CD3/CD28/CD2 activator beads were included (StemCell Technologies). In parallel, 10 nm of ibrutinib (Tocris) together with 1 nm of rapamycin (Tocris) were administered during T cell expansion. Media, drugs and activation beads were replenished every 5 days. After three weeks, cells were collected and stained in buffer containing Fc block (eBioscience) with the following fluorescently conjugated human monoclonal antibodies against CD45, CD3, CD4, CD8, CD45RO, CD45RA, PD1, HLA-DR, CD107a, TOX, CXCR5, and CTLA4 (see Table S2). Cell acquisition was performed using a BD FACS LSRII SORP (BD Biosciences). A minimum of 5 x 10^5^ events was collected and analyzed using FlowJo ver10.7 (Tree Star). Total RNA was extracted from expanded TILs using RNeasy Mini Kit (Qiagen) following the manufacturer's protocol. RNA was quantified using NanoDrop NF10000 spectrophotometer (Thermo Fisher Scientific). 50 ng of total RNA was used for gene expression analysis with the NanoString nCounter PanCancer IO 360™ gene expression panel (NanoString Technologies), which contains a pool of 770 human synthetic oligonucleotides (including internal reference genes). For analysis, unsupervised hierarchical clustering analysis shows the top downregulated genes in post-treated samples, compared with matched pre-treated ones. Data analyses were performed using R 'pheatmap' package (ward.2 method). The heatmap legend represents the foldchange. Comparison of the indicated pathway signature scores between pre- vs. post-treated MPM samples based on Nanostring data. The genes in each pathway across all 12 samples were globally normalized/scale, then subjected to calculate the signature score based on the sum of the expression of genes in each pathway. The statistical significance was calculated by the student's two-tailed paired t-test. Data analyses were performed using R v.3.6.1.

### Statistical analysis

Data are expressed as mean ± SD. Comparisons between two groups were carried out using the parametric student's two-tailed paired or unpaired t-test for normally distributed data. If data were not distributed normally, a non-parametric Wilcoxon rank-sum test (for unpaired) or Wilcoxon signed-rank test for paired samples was used between the two groups. One-way analysis of variance (ANOVA) followed by Newman-Keuls post hoc test was used for analysis of more than two groups. The numbers of samples (biological replicates) per group (n), or the numbers of experiments (technical replicates) are specified in the figure legends. Data were analyzed using GraphPad Prizm 9 software or R Statistical Computing environment v3.6.0 (http://www.r-project.org).

## Results

### ECM/stromal and IA gene profiles in MPM patients are linked with survival

**Figure 1:**
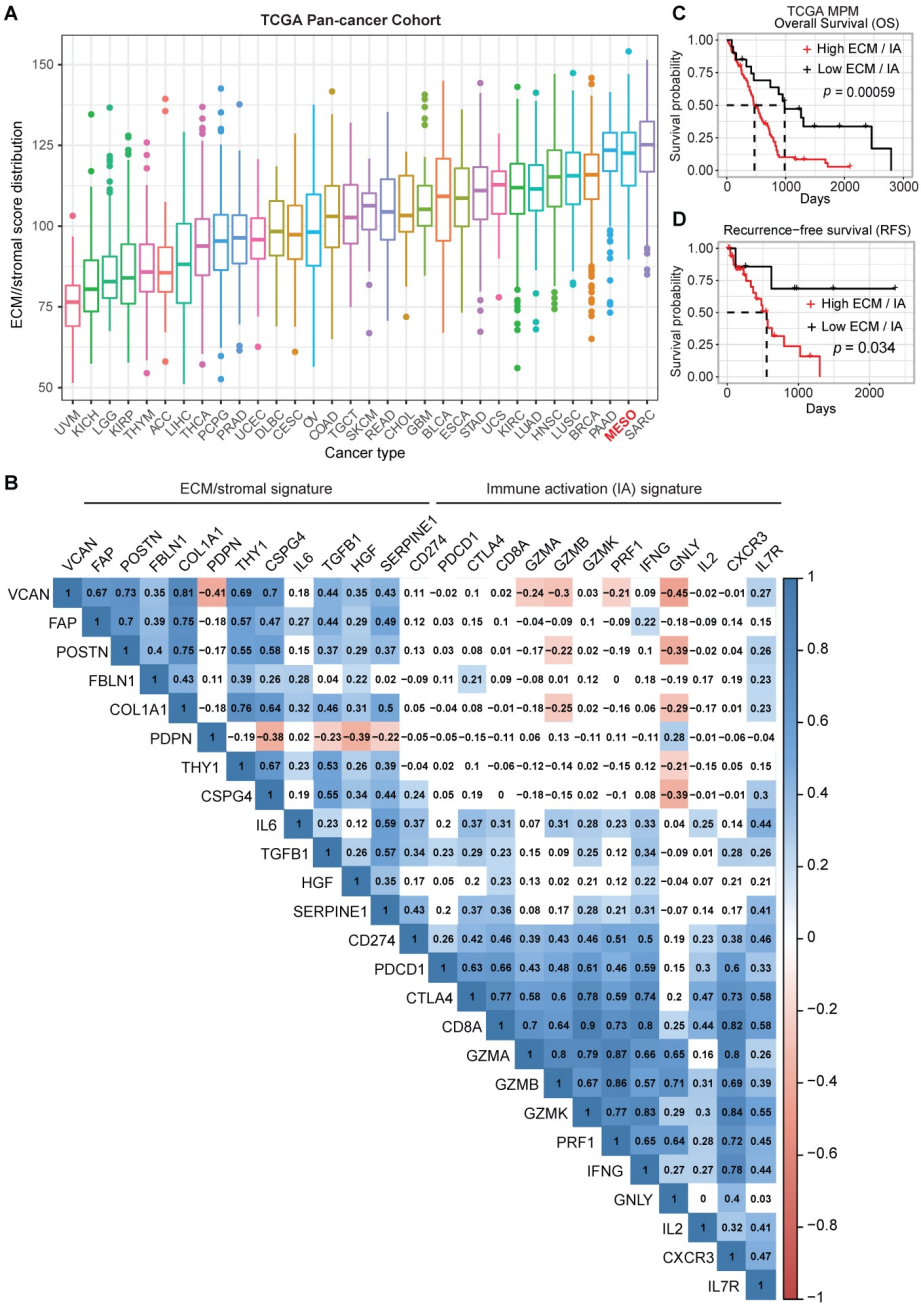
** ECM/stromal and CD8 T cell-derived immune activation gene signatures are associated with poor prognosis in MPM**. (**A**) Boxplots showing extracellular matrix (ECM)/stromal signature scores across various cancer types in TCGA. (**B**) Correlation analysis of the individual genes in ECM/stromal and immune activation (IA) gene sets across the TCGA MPM cohort. Transcriptomic data of treatment naïve MPM patients were obtained from TCGA (https://portal.gdc.cancer.gov/projects/TCGA). The numbers in the correlogram indicate the correlation coefficient (Spearman). Significant positive (in blue) and negative (in red) correlations are shown, with color intensity proportional to the correlation coefficient. Non-significant correlations retain a blank background. The *p*-value < 0.05 is considered significant. (**C-D**) Unadjusted Kaplan-Meier curves showing overall survival (OS) (**C**) and recurrence-free survival (RFS) (**D**) by the ratio of ECM/stromal to IA gene expression in treatment naïve MPM.

The importance of the mesenchymal fraction within the stromal compartment contributing to cancer progression and failure to respond to ICB has been shown for several solid tumors 6. However, there is a paucity of data on the role of the mesenchymal fraction in MPM progression and response to therapy. To further understand the importance of the stromal elements of the TME in MPM, we assembled an ECM/stromal gene signature using defined genes previously shown to be expressed predominantly in stromal rather than cancer cells in solid tumors 6, 17, 18. We combined this with a CD8 IA signature^19^, ^20^ and applied both signatures to the TCGA MPM cohort. Our analysis revealed that MPM displays a high ECM/stromal gene signature, compared with many other solid tumors (Figure 1A), suggesting an enrichment of TME in MPM tumors. A mutually positive correlation among the ECM/stromal genes was observed in MPM (Figure 1B). There also was a mutually positive correlation among the IA-relevant genes (Figure 1B), but ECM/stromal gene signature correlated negatively with IA signature. Notably, the expression of cytotoxic granules granzyme B (GZMB), granzyme A (GZMA) and granulysin (GNLY), expressed primarily by conventional CD8+ T cells, were most negatively correlated with the ECM/stromal gene signature (Figure 1B). Importantly, a low ECM/stromal gene signature was associated with a longer overall and progression free survival (Figure S1A-B). In contrast, a low IA gene signature was associated with shorter overall and recurrence free survival (Figure S1C-D). A high ratio of ECM/stromal to IA gene signature score translated into poor overall and recurrence free patient survival (Figure 1C-D). There was no correlation between the IA and ECM gene signatures with commonly reported mutations in MPM (Figure S1E). Taken together, these data showed that similar to an EMT signature, a high ECM but low IA gene signature defines an aggressive phenotype of MPM, suggesting that the enriched TME may contribute to an ineffectual host immune response.

### MPM is infiltrated by PD1^hi^ TILs

**Figure 2:**
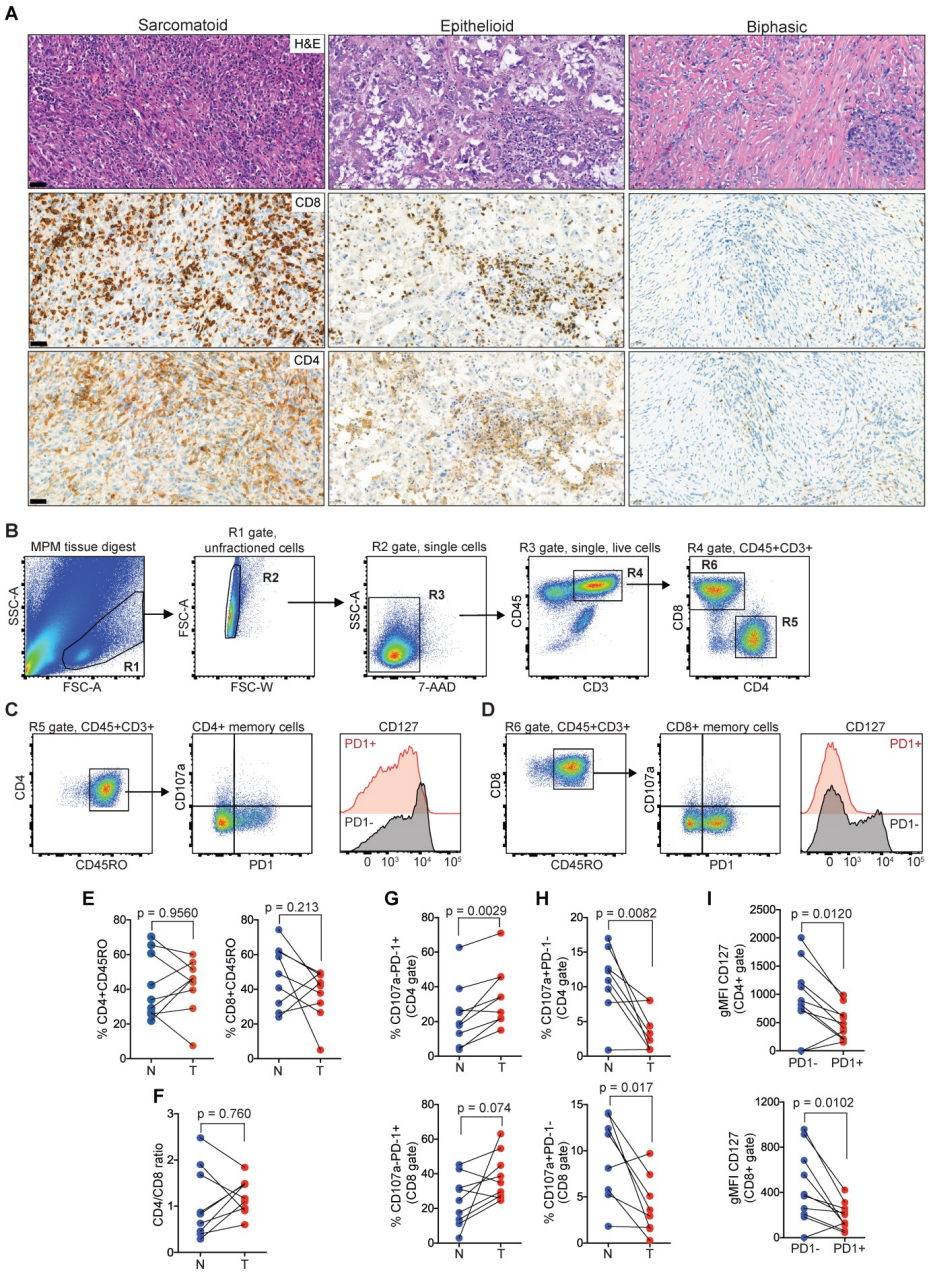
**Memory CD4 and CD8 TILs in MPM overexpress PD1 while downregulated CD127.** (**A**) Representative MPM specimens showing differences in distribution of TILs in serial histological sections (scale bar: 50 µm; H&E, CD8, CD4). (**B-D**) Bivariate plots showing gating strategy to select single live T cells (**B**) and co-expression of PD1, CD107a and CD127 in CD4 (**C**) and CD8 (**D**) memory compartments. (**E-F**) Scatter plots showing the frequency of CD4 and CD8 TILs (**E**) and CD4/CD8 ratio (**F**). (**G-H**) Scatter plots showing the frequency of CD4^+^ (top) and CD8^+^ TILs (bottom) subgated for PD1 and CD107a. (**I**) Scatter plots of CD4 (top) and CD8 memory TILs (bottom) showing geometric mean fluorescence intensity (gMFI) of CD127 in cell subsets subgated based on PD1 expression in MPM. All data in **E-I** determined by flow cytometry. Significant differences in **E-H** calculated between matched tumor (T) and non-adjacent lung control tissue pairs (N) in 9 patients using Wilcoxon signed-rank test. All tests were two-tailed. See also Figure S2 in the online data supplement for full gating strategy.

Next, we sought to characterize TILs in clinical MPM samples as the negative correlation of cytolytic effector molecules (GZMB, GZMA, GNLY) with the enriched ECM/stromal gene signature, suggested an impairment in effector function concomitant with high ECM/stromal microenvironment (Figure 1B). In an MPM patient cohort undergoing tumor resection, the number of CD4 and CD8 TILs varied between tumors (Figure 2A). Examining the TIL fractions using flow cytometry (Figure 2B-D, and Figure S2), there was no difference in the number of CD45RO+CD4+ (tumor, 42.2 ± 15.8 versus normal, 41.87 ± 18.8) or CD45RO+CD8+ memory T cells (tumor, 37.2 ± 14.4 versus normal, 47.8 ± 17.9) in MPM compared with matched, nonadjacent uninvolved lung tissue (Figure 2E). This coincided with a lack of change in the CD4/CD8 ratio (Figure 2F). Despite this, PD1 was upregulated on the surface of both CD4 (tumor, 35 ± 17.2 versus normal, 23.6 ± 18) and CD8 memory T cells (tumor, 38.2 ± 13.5 versus normal, 24.6 ± 14.5) in MPM versus normal lung control, which coincided with downregulation in the expression of the degranulation marker CD107a in both CD4 (tumor, 3.6 ± 3 versus normal, 10.9 ± 5) and CD8 memory T cells (tumor, 4 ± 3.2 versus normal, 9.2 ± 4.6) (Figure 2G-H). Surface expression of CD127 (IL7R), a homeostatic surface receptor and marker of long-living memory T cells 32, was also diminished on CD4+PD1+ (PD1+, 486 ± 288 versus PD1-, 923 ± 643) and CD8+PD-1+ memory TILs (PD1+, 188 ± 119 versus PD1-, 451 ± 320) in MPM (Figure 2I). Collectively, these data suggest TIL function may be impaired, despite their ability to infiltrate into the MPM tumor compartments.

### Chemotherapy shapes tumor immunity in a subset of MPM

**Figure 3:**
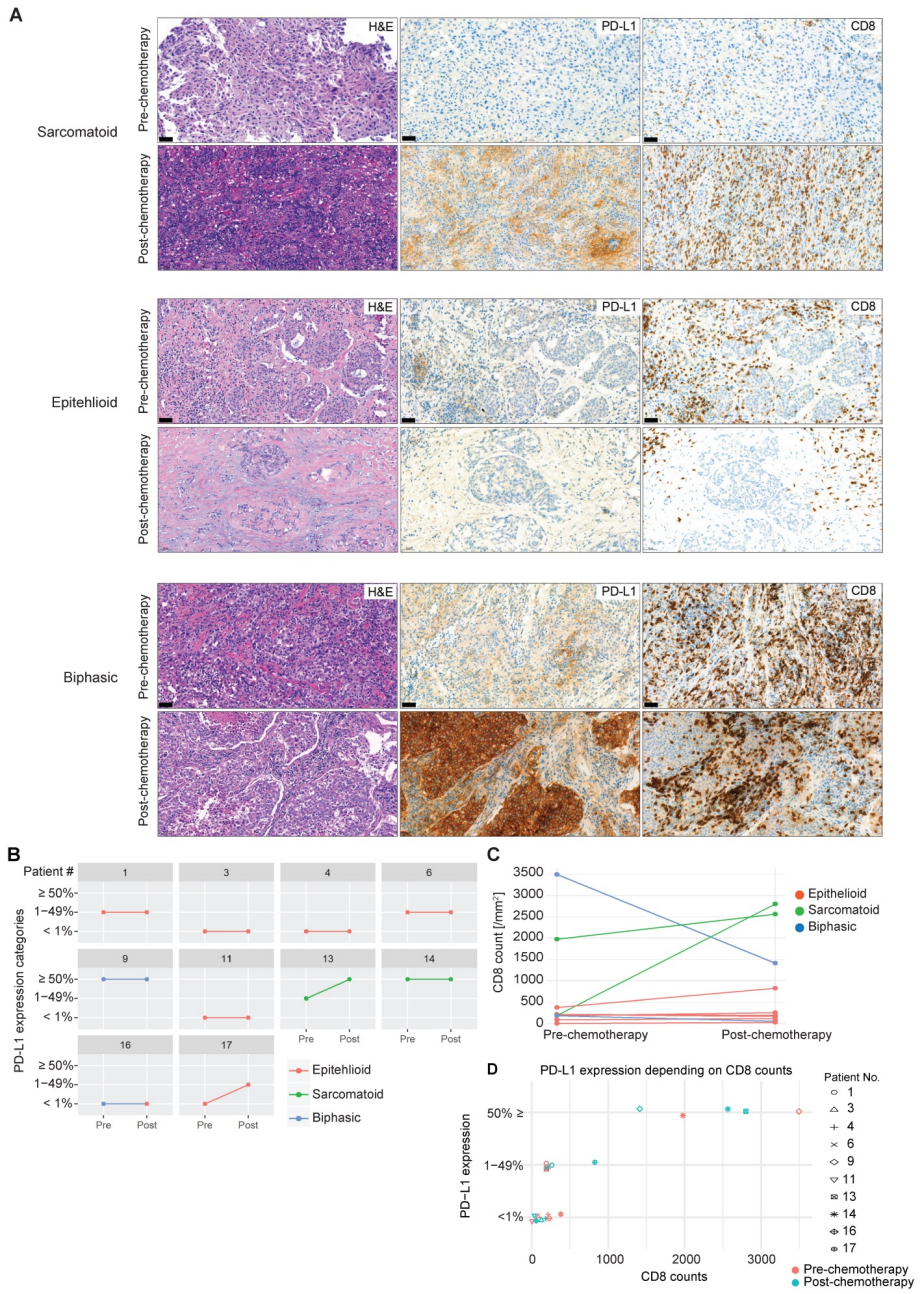
** PD-L1 expression and CD8 TIL infiltration after chemotherapy**. (**A**) Representative MPM specimens were analyzed for H&E, PD-L1 and CD8 expression in serial sections before and after chemotherapy. (**B**) Scatter plots showing the change in PD-L1 in response to chemotherapy in matched patients. (**C**) Scatter plots showing the change in CD8 infiltration in response to chemotherapy in matched patients. (**D**) Scatter plots showing the correlation between PD-L1 intensity and CD8 infiltration. n = 10. Significant differences in **C** calculated between pre-chemo and post-chemo tissue pairs using Wilcoxon signed-rank test. All tests were two-tailed.

Whether exposure to chemotherapy, the standard first-line treatment in MPM patients 33, is involved in promoting tumor immunity via an increase in infiltration of activated CD8 T cells and upregulation of PD-L1 is not known. In selected patients (n = 10), we were able to investigate CD8 TIL distribution and frequency and expression of PD-L1 before and after chemotherapy (Figure 3A). Upregulation of PD-L1 represents a critical pathway used by solid tumors to escape the immune system by binding to PD1 antigen expressed on activated TILs, inducing their negative regulation. We found an increase in tumor cell PD-L1 expression post-chemotherapy in two out of ten patients (Figure 3B). This lack of response might be due to the small sample size and confirmation warrants a larger collection. Of note, sarcomatoid MPM (n = 2), a histological subtype that has more mesenchymal stromal compartments and predicts poorer prognosis 34, has higher basal expression of PD-L1, and chemotherapy induced PD-L1 upregulation in one sarcomatoid tumor with lower basal PD-L1 expression (Figure 3B). Similarly, an increase in CD8 TIL infiltration was observed in the two cases of sarcomatoid MPM, contrary to epithelioid MPM (Figure 3C), which might be due to the enriched immune microenvironment in sarcomatoid MPM 35, 36. In line with these observations, high PD-L1 expression (≥ 50%) was associated with higher CD8 counts (Figure 3D). Overall, neoadjuvant chemotherapy had a heterogeneous effect on tumoral PD-L1 expression and CD8 number in a subset of MPM, which might be related to the histological subtypes that differ in the enrichment of stromal compartments.

### CD90+ cells overexpressing PD-L1 comprise a key source of the ECM/stromal gene signature

The stromal compartment, consisting primarily of CAFs and pericytes, strongly influences the progression of solid tumors 37. Recently, a population of circulating mesothelial precursor cells (MPCs) expressing the membrane glycoprotein CD90 (THY-1), which was also included in the established ECM/stromal gene signature, was shown to be associated with pleural damage and recruitment of inflammatory leukocytes in MPM patients 38. In TCGA MPM samples, there was a strong positive correlation between CD90 and ECM/stromal gene signature (Figure S3A). Clinically, univariate and multivariate analyses showed that CD90 expression was an independent factor predicting poor overall and recurrence-free survival (Figure S3B-E).

**Figure 4:**
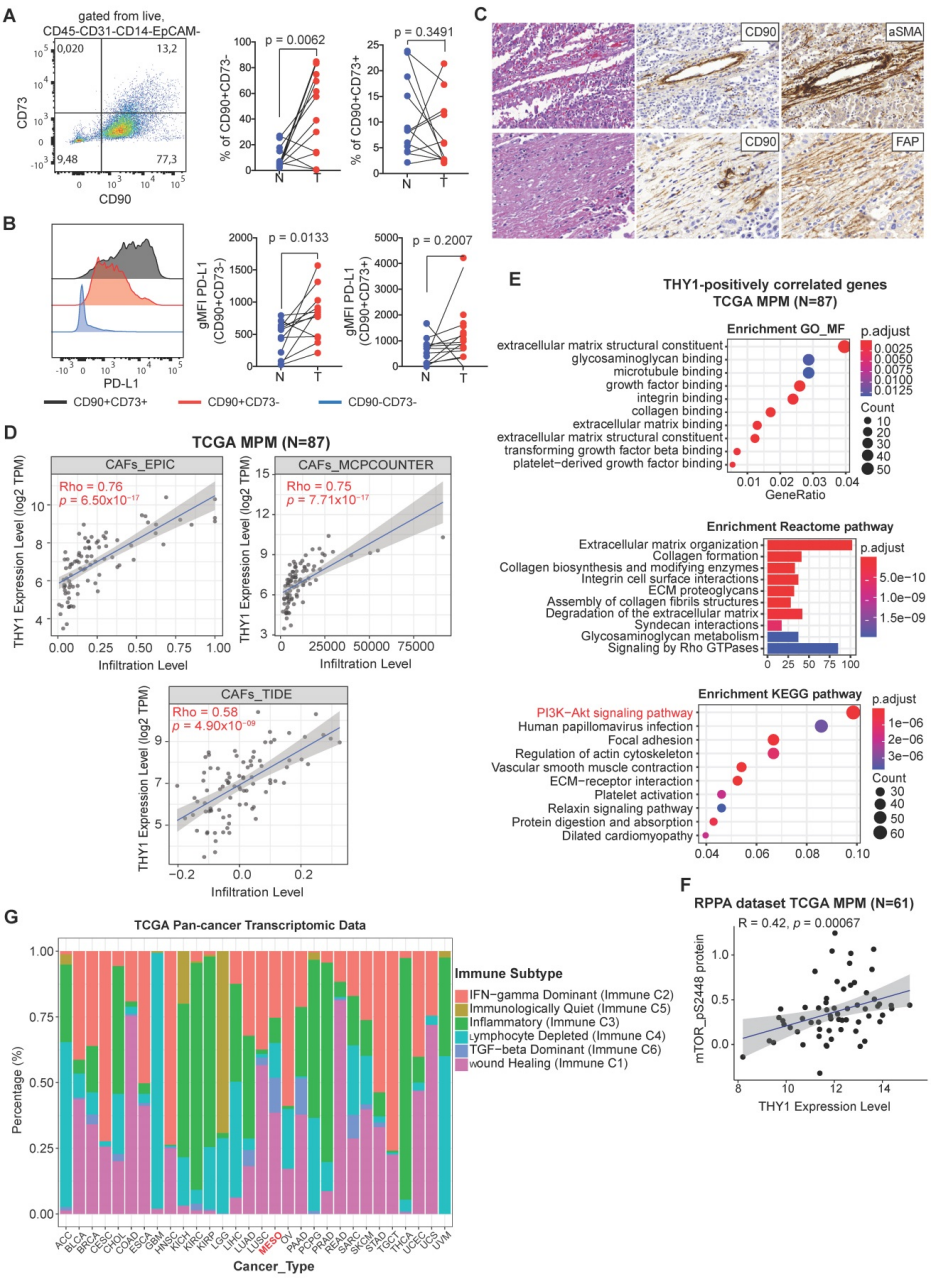
** Characterization of CD90+ mesothelial precursor cells in MPM.** (**A**) Representative bivariate plot of mesenchymal fraction (CD45-CD31-CD14-EpCAM-) subgated onto bivariate plots for CD73 and CD90 (left panel). Scatter plots (right panels) of subsets of EpCAM^-^ cells subgated based on CD90 and CD73. (**B**) Representative histogram showing PD-L1 expression (left panel) in various subsets based on CD90 and CD73 expression. Scatter plots (right panels) showing gMFI for PD-L. (**C**) Representative IHC of post-neoadjuvant chemotherapy (NAC) MPM sample in hematoxylin & eosin (H&E), as well as co-staining with CD90 and αSMA (top panels) and CD90 with FAP (bottom panels). **D**) Correlative analysis (purity-adjusted spearman's R) between CAFs (cancer-associated fibroblasts) signature and THY1 (encoding CD90) gene expression across TCGA MPM tumor samples. CAFs' abundance was estimated by multiple immune deconvolution methods: EPIC, MCPcounter, or TIDE algorithms. (**E**) Functional enrichment (top 10) analysis of genes that were significantly positively correlated with THY1 expression across TCGA MPM tumor samples. KEGG (Kyoto Encyclopedia of Genes and Genomes) pathway, Gene Ontology (GO) Molecular Function (MF) and Reactome enrichment analyses were performed using ClusterProfiler package in *R*. (**F**) Correlative analysis (spearman's R) between phospho-mTOR (S2448), a biomarker indicating the activation of mTOR signaling and THY1 (encoding CD90) gene expression across TCGA MPM tumor samples. Protein quantification of phospho-mTOR (S2448) was based on RPPA dataset from TCPA portal (http://tcpaportal.org/tcpa/). (**G**) Bar graphs showing distribution of Immune subtypes (C1-C6) within TCGA tumor types including MPM based on the immune subtype models by Thorsson et al. 2018 23.

To investigate the presence of mesenchymal cells in MPM tissue, we applied multiparametric flow cytometric analysis to freshly digested MPM tissue using a multicolor antibody panel. Within MPM, we could identify two main population clusters based on differential expression of CD90 and CD73, an ecto-5'-nucleotidase that serves as an important checkpoint by generating the immunosuppressive molecule adenosine 39 that is upregulated on CAFs 40 (Figure 4A). After gating out hematopoietic (CD45, CD14) and endothelial cells (CD31) (see Figure S4A for full gating strategy), the predominant mesenchymal cluster was single positive for CD90 (tumor, 44.2 ± 32 versus normal, 10.3 ± 8.8, n = 12) (Figure 4A). There also was a population that co-expressed CD73 (tumor, 5.8 ± 6.7, n = 12); however, this did not differ from uninvolved normal tissue (8.6 ± 7.7, n = 12) whereas single CD73 cells were rare. In addition, we showed that CD90+CD73- cells in MPM were enriched in PD-L1 expression (tumor, 802 ± 393 versus normal, 431 ± 274, n = 12) (Figure 4B).

CAFs represent a heterogeneous population characterized by different markers 9, 10. Additionally, to know the potential identity of CD90+ mesenchymal cells, we evaluated the co-expression of CD90/αSMA and CD90/FAP, two classical CAF markers, on sequential sections of three patients (epithelioid, sarcomatoid and biphasic malignant mesothelioma) including pre-neoadjuvant chemotherapy (NAC) as well as post-NAC samples. Almost all CD90+ cells could be seen to co-stain with αSMA and FAP (Figure 4C and Figure S5; Table S4). In agreement, based on three different algorithms, we observed a positive correlation between THY1/CD90 gene expression and a CAF gene signature across TCGA MPM tumor samples (Figure 4D). Moreover, THY1/CD90 positive MPM tumors were functionally enriched in genes associated with microenvironment sensing such as the extracellular matrix, integrin binding and collagen organization (Figure 4E). Interestingly, genes in the PI3K-Akt signaling pathway represented the top positively pathway correlated with THY1/CD90 gene expression across TCGA MPM cohort (Figure 4E). Further analysis at the protein level demonstrated a positive correlation between phospho-mTOR (S2448), a key downstream effector of PI3K-Akt signaling and a biomarker indicating the activation of mTOR signaling, and THY1 (encoding CD90) gene expression across TCGA MPM tumor samples (Figure 4F). Furthermore, it has been reported that tumor cells overexpress CD90, which binds to Mac-1 (ITGB2), an integrin binding proein expressed on neutrophils and promote tumor metastasis via a CD90-TIMP1 loop 41. Thus, to investigate whether Mac-1 or TIMP1 levels change in samples with high CD90+ stromal cells in MPM, we performed a correlation analysis and found that there was no correlation between THY1, ITGB2, and TIMP1 expression in MPM (Figure S6). Together, CD90+ mesenchymal cells may mark a major CAF population with activated PI3K-Akt-mTOR signaling in MPM.

Given the differential expression between tumors and adjacent normal tissue, next, we sought to understand the function of the CD90+CD73- cell subset in MPM. We generated a panel of 12 patient-derived CD90+CD73- MPM cell lines using prospective FACS (Figure S4A, B). At the protein level, CD90+CD73- cells express several markers known to be expressed by mesenchymal cells, as well as several toll-like receptors (TLRs) (Figure S4C). At the mRNA level, we showed that the primary cultures of patient-derived CD90+ MPM cells including all histological subtypes are enriched in ECM/stromal genes that were found to correlate with poor overall survival in the TCGA MPM cohort, which include FAP, versican (VCAN) and periostin (POSTN), a TGFβ superfamily-responsive matricellular protein (Figure S4D). Moreover, we found that CD90+CD73- MPM cells were resistant to the cytotoxic effects of pemetrexed/cisplatin exposure (Figure S4E). In line with our findings, MPM is enriched in a TGF-beta dominant immune subtype compared with other solid tumors (Figure 4G), which were characterized by a dominant TGF-β1 gene signature and shown to be associated with poor overall survival 23. Collectively, CD90+CD73- MPCs, overexpressing PD-L1 and predicting poor prognosis, comprise a key source of the ECM/stromal gene signature in MPM tumors.

### CD90+ MPC cells negatively regulate the functional response of T cells

**Figure 5:**
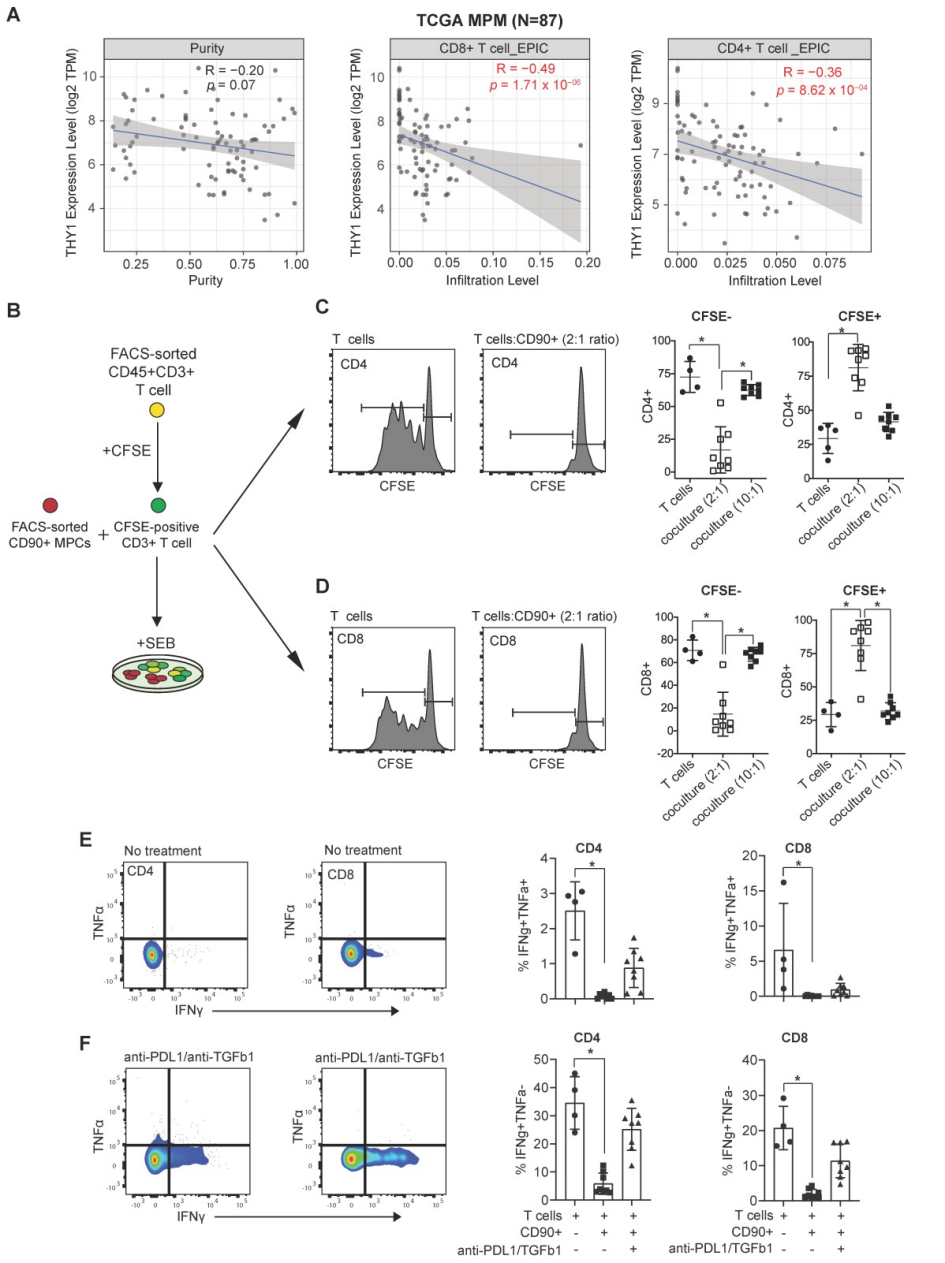
** Immunosuppressive function of CD90+ MPC cells.** (**A**) Correlative analysis (purity-adjusted spearman's R) between immune infiltration estimation (CD4+ or CD8+ T cells) and THY1 (encoding CD90) gene expression across TCGA MPM tumor samples. Immune infiltration profiles were estimated by EPIC algorithm 22. Data were from TIMER2.0 (http://timer.comp-genomics.org/), a comprehensive resource for systematical analysis of immune infiltrates across TCGA cancer types. (**B**) Schematic showing coculture experimental setup. (**C, D**) Representative flow cytometric histograms showing proliferation of CFSE-labeled CD4 (**C**) and CD8 T cells (**D**) isolated from the peripheral blood of a healthy donor 5 days following activation with staphylococcal enterotoxin B (SEB, 500 ng/mL) alone or in the presence of immune primed (IP) CD90+ MPCs cells at a 2:1 ratio. Scatter plots showing proliferation of CFSE-labeled CD4 and CD8 T cells cocultured with vehicle or IP CD90+ MPCs. For coculture conditions (n = 8) in total. (**E, F**) Representative bivariate plots showing TNFα and IFNγ expression in CD4 (**E**) and CD8 T cells (**F**) following activation with SEB (500 ng/mL) in the presence of IP CD90+ MPCs treated with vehicle (No treatment) or 10 ug/ml of anti-PD-L1 and 10 ug/ml of anti-TGF-β1. Scatter plots showing % of IFNγ+/TNFα+ and IFNγ+/TNFα- in CD4 and CD8 T cells. Data in C-F determined by flow cytometry and presented as mean ± SD. Significant differences calculated using one way ANOVA followed by post hoc Tukey's range test.

We next aimed to assess whether CD90+ MPM cells possess an immunosuppressive phenotype. First, we used a computer-based tool EPIC (Estimating the Proportion of Immune and Cancer cells) 22 to determine the correlation between THY1/CD90 expression and immune infiltration. We observed that there was a negative correlation between THY1/CD90 mRNA expression and the infiltration of both CD8 and CD4 TILs in MPM using TCGA MPM cohort data (Figure 5A). Next, we cultured CD90+ MPM cells together with CD3+ T cells isolated from peripheral blood of healthy donors (Figure 5B). We showed that CD90+ MPCs cells immune primed with TNFα and IFNγ inhibited the stimulation of both CD8+ and CD4+ T cells in response to TCR-dependent Staphylococcal enterotoxin B (SEB) activation (Figure 5C-D and see Figure S7A-C) and decreased CD107a expression (Figure S7D-E). Treatment with anti-PD-L1 and anti-TGFβ1 neutralizing antibodies was effective in increasing the secretion of IFNγ in both CD8 and CD4 T cells in the presence of CD90+ MPCs (Figure 5E-F). Together, these data highlight that MPM-derived CD90+ cells within the TME represent the major subset that potentially contributes to the immunosuppressive TME within MPM tumors.

### MPM TILs maintain the ability to expand in a TCR-dependent manner and are reprogrammed in the presence of Ibrutinib and Rapamycin

In melanoma and NSCLC, ICB targeting the immune checkpoint proteins PD-L1 and CTLA4 have resulted in improved patient survival due to reinvigoration of exhausted T cells. However, in MPM, the response rates to single-agent ICB have failed to improve patient survival 15. Whether TIL exhaustion in MPM is behind the failure to respond to ICB remains unresolved. One of the confounding factors might be the presence of an immunosuppressive TME, which represents a physical and chemical barrier to proper host immune function 37.

**Figure 6:**
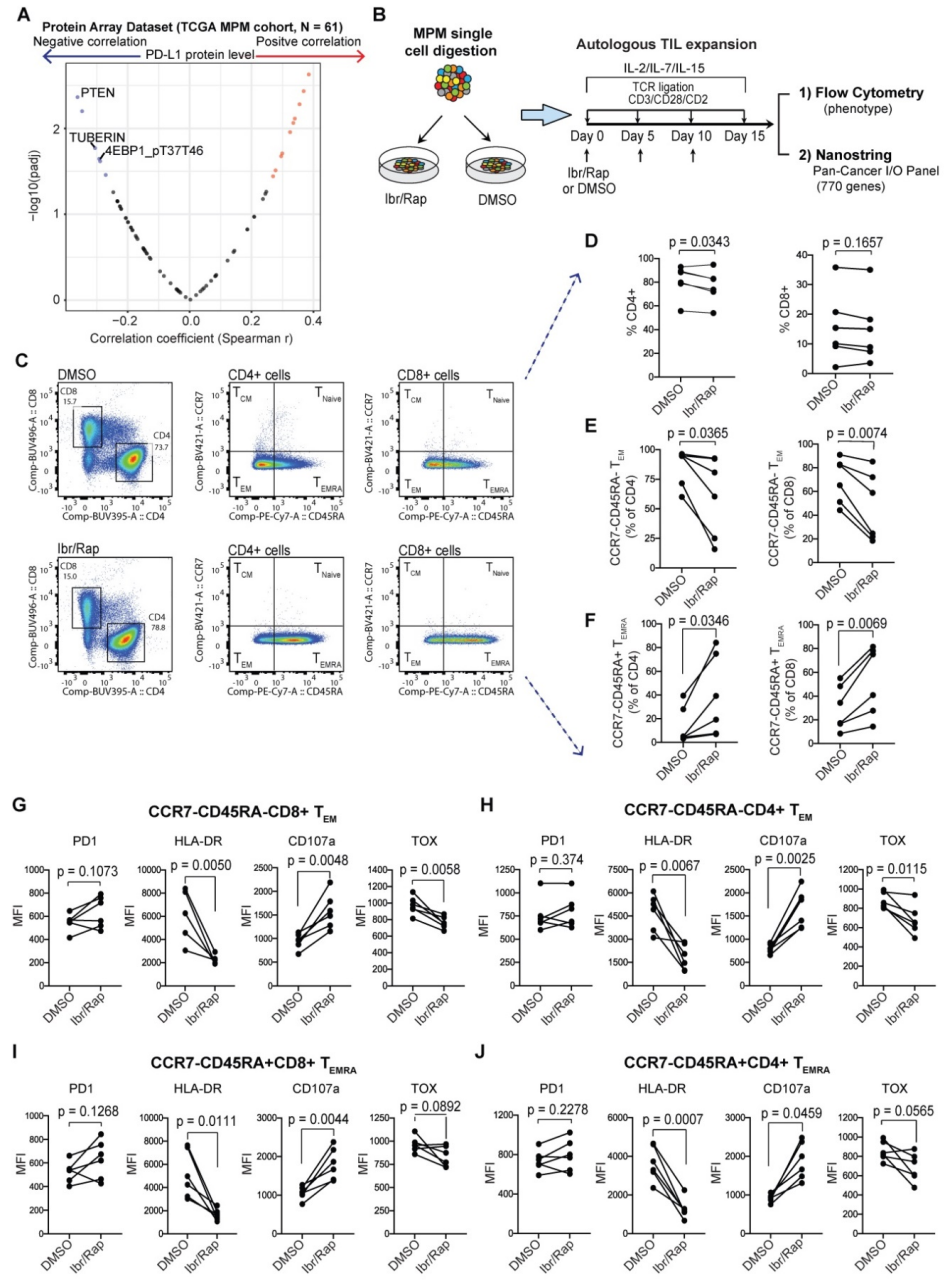
** Plasticity of MPM TILs**. (**A**) Protein array dataset from TCGA MPM cohort showing proteins that are significantly correlated with PD-L1 protein expression. The plot shows proteins that negatively regulate PI3K-mTOR pathway are most enriched. (**B**) Graphical overview showing methodology of the experimental design used to expand T cells from digested MPM tumor samples, n = 8, biological replicates. (**C**) Representative bivariate flow plots comparing T cells subsets based on CCR7 and CD45RA expression in CD4 and CD8 T cells in DMSO (top panels) compared with Ibr/Rap (Ibrutinib and rapamycin) (bottom panels) treated samples in a matched patient. (**D-F**) Scatter plots showing change in frequency of CD4 and CD8 T cells (**D**), as well as CCR7^-^CD45RA^-^T_EM_ (**E**), and CCR7^-^CD45RA^+^ T_EMRA_ subsets (**F**) in CD4 and CD8 TILs treated with DMSO compared with Ibr/Rap. **(G-J)** Scatter plots showing change in frequency in several markers in both CD8 (**G**) and CD4 (**H**) CCR7^-^CD45RA^-^T_EM_ subsets, and CD8 (**I**) and CD4 (**J**) CCR7^-^CD45RA^+^ T_EMRA_ subsets. biological replicates (n = 8). Ibr/Rap, Ibrutinib (10 nm), Rapamycin (1 nm).

Our above evidence demonstrated that CD90+ MPCs played a key immunosuppressive role within the TME of MPM tumors and therefore represented a potential therapeutic target. Molecularly, these cells were characterized by activated PI3K-mTOR pathway (Figure 4E). Moreover, we found that proteins negatively regulating the PI3K-mTOR pathway significantly negatively correlated with PD-L1 protein expression (Figure 6A), suggesting that activated PI3K-mTOR pathway may modulate host anti-tumor immunity in MPM 3. Therefore, inhibitors targeting PI3K-mTOR pathway might enhance the anti-tumor immunity in MPM. First, we assessed whether TILs were amenable to TCR-dependent expansion in culture in the absence of the TME. Second, we also investigated whether TIL phenotype could be improved using a selective inhibitor of the mTOR pathway, Rapamycin together with Ibrutinib, a dual inhibitor of Bruton's tyrosine kinase and IL-2-inducible T-cell kinase (ITK), which has been shown to alter CAR T cells during their expansion period improving their function 42, 43.

MPM samples digested to single cells were cultured *ex vivo* in the presence of gamma chain cytokines IL-2, IL-7 and IL-15 with repeated TCR-dependent ligation using CD3/CD28/CD2 beads in the absence or presence of Ibrutinib (10 nM) and low dose Rapamycin (1 nM) (herein referred to as Ibr/Rap) (Figure 6B). Using multiparametric flow cytometry (Figure 6C), we showed that expanded TILs in the presence of Ibr/Rap had a small but significant decrease in the CD4 compartment but not the CD8 compartment (Figure 6D). Despite this, there was a significant shift in both CD4 and CD8 TILs away from effector memory (T_EM_) (CCR7-CD45RA-) and toward TILs that re-express CD45RA (CCR7-CD45RA+) termed T_EMRA_ (Figure 6E, F). Interestingly, there was a trend of increase in PD1 expression in both CD4 and CD8 T_EM_ and T_EMRA_ subsets (Figure 6G-J) but this was not significant. However, we noted that the treatment with Ibr/Rap resulted in downregulation of HLA-DR, whereas the marker of T cell degranulation CD107a was upregulated (Figure 6G-J). Importantly, these changes coincided with decreased expression of the transcription factor TOX (thymocyte selection-associated HMG box), a critical marker of exhausted human effector memory CD8 T cells 44.

**Figure 7:**
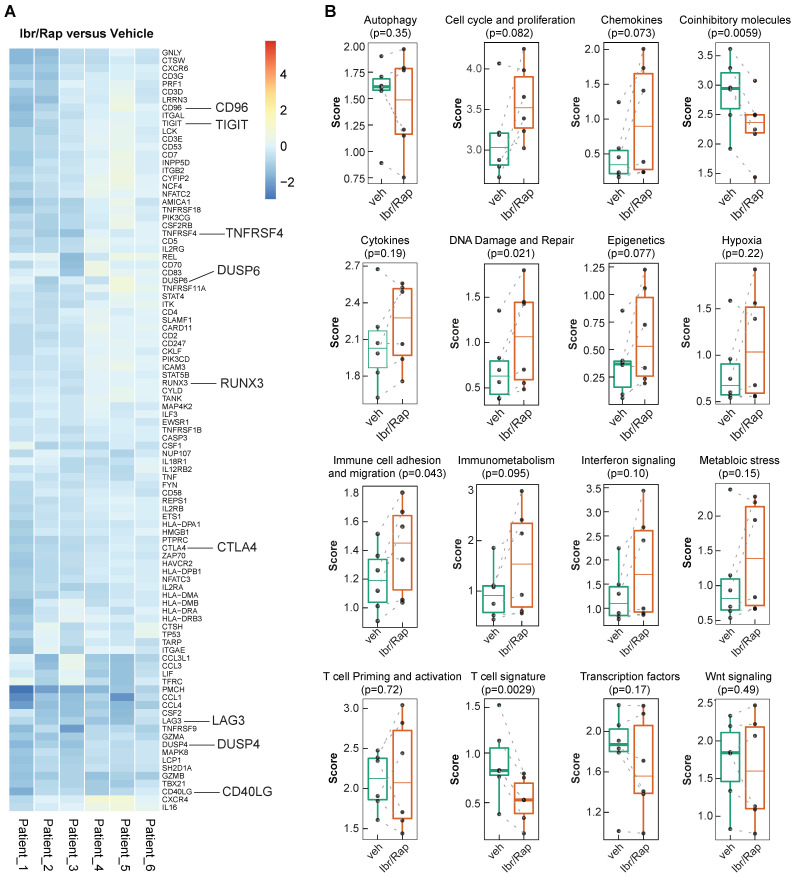
** Gene signature development in *ex-vivo* expanded TILs treated with Ibrutinib and rapamycin.** (**A**) Heatmap of the top ranked genes expressed in TILs following expansion *ex vivo* in response to chronic TCR-dependent activation in the presence of Ibrutinib and rapamycin (Ibr/Rap) VS. DMSO vehicle. Each column represents an individual patient. (**B**) Box plots comparing the change in gene signature pathways between Ibr/Rap and Vehicle (DMSO) treated groups.

Given that Ibr/Rap skewed CD8 and CD4 TILs towards a T_EMRA_ phenotype, we analyzed gene expression profiles of expanded TILs using a custom panel of over 700 tumor- and immune-related genes on the NanoString nCounter PanCancer Immune profiling platform (NanoString Technologies Inc.). We showed that TILs expanded in the presence of Ibr/Rap downregulated several co-inhibitory molecules consisting of LAG3, TIGIT, CTLA4, CD96 and DUSP4 (Figure 7A), which coincided with a decrease in the co-inhibitory molecule and T cell signature pathways (Figure 7B). In contrast, TILs expanded in the presence of Ibr/Rap showed an increase in signature pathways associated with DNA damage and repair, and immune cell adhesion and migration (Figure 7B). Moreover, there was a trend of increase in epigenetic, cell cycle and proliferation and immunometabolism signatures in expanded TILs in the presence of Ibr/Rap (Figure 7B). Taken together, these data demonstrated that TILs infiltrating MPM tumors preserve their ability to respond in a TCR-dependent manner and *in vivo* their decreased activation may be associated with an immunosuppressive TME. Therefore, an autologous, TIL-based adoptive strategy involving combined ITK (interleukin-2 inducible kinase, e.g. Ibrutinib) plus mTOR pathway (e.g. Rapamycin) tuning may represent a novel treatment for MPM patients.

## Discussion

In the present work, we showed that patients' MPM was endowed with a population of CD90+ MPM cells, which enrich an ECM/stromal gene signature and possess immunosuppressive properties and confer resistance to chemotherapy. These changes coincided with the presence of PD1^hi^ CD4+ and CD8+ memory TILs with downregulated expression of CD127, a marker associated with enhanced memory stem cell-like function. Despite this, CD8+ and CD4+ memory TILs exhibited *ex vivo* expansion capability following repeated TCR-dependent stimulation and the presence of Ibr/Rap significantly skewed TIL phenotype towards T_EMRA_ TILs while downregulating co-inhibitory molecules and molecules of exhaustion and alteration of several gene signatures of immune function. Based on these findings, we conclude that CD90+ MPM cells represent a major subpopulation constructing an immunosuppressive TME, and targeted reprogramming of the TME represents an attractive strategy to improve TIL function in MPM.

The tumor stroma is an important organizer and potential therapeutic target in solid tumors 45. Tumor stromal elements consisting of CAFs and cancer-associated pericytes are the major source of the EMT- and stroma-related gene expression signatures that predict resistance to PD1 blockade across a wide variety of solid tumors 6, 18. Whether the tumor stroma exerts a similar role in MPM has not been deeply examined. MPM is associated with EMT 3, 4 and the potential clinical benefit from single-agent ICB targeting PD1 remains unresolved in MPM patients 15. One particularly important marker expressed by mesenchymal cells within the tumor stroma is the cell surface marker CD90 (THY1) 46. CD90+ cells have previously been identified in MPM 47. Recently, Kelley and colleagues 38 identified subpopulations of circulating mesothelial precursor cells based on the expression of mesothelin, CD90 and CD34 that lacked the pan-hematopoietic marker CD45 thereby ruling out circulating fibrocytes. The authors showed that circulating mesothelin+CD90+CD34- cells were associated with exposure to asbestos and have been implicated in pleural damage. Moreover, high circulating mesothelin+CD90+CD34- cell subpopulations were associated with wider surgical resection. It was previously shown that CD90 expression was associated with acquired resistance in primary MPM cultures and correlated with increased expression of lncRNA RP11-334E6.12 48. Importantly, CD90^high^ expression levels are associated with poor overall survival in the TCGA MPM patient cohort. Tumor-infiltrating mesenchymal stromal cells are immunoregulatory, targeting both the innate and adaptive immune system 37. Importantly, earlier work demonstrated that the heme-containing enzyme indoleamine 2,3-dioxygenase (IDO) was dispensable for the immunosuppressive function of IFNγ-primed mesenchymal stromal cells 49. Interestingly, we observed that only immune primed CD90+CD73- MPM-derived cells were able to suppress both CD4 and CD8 T cells. Whether these cells are related to a subset of CD90+ mesenchymal cells we recently observed within postnatal human lung with immunosuppressive properties is not known 29. Further studies are required to examine the molecular mechanisms whereby CD90 is directly involved in malignant progression and whether targeting their immunosuppressive properties translates into enhanced host immune function in MPM.

In solid tumors, the presence of infiltrating CD8+ cytotoxic T cells is a positive prognostic indicator 19. Biopsies taken from MPM patients indicated that tumors are infiltrated by immune effector cells. However, this is counterbalanced by cytokines and T regulatory cells that contribute to an immunosuppressive microenvironment 50. Recently, CD8+ TIL hypofunction was demonstrated in MPM patients 51. TME enriched in cytotoxic T lymphocytes is associated with tumor expression of PD-L1, poor response to chemotherapy and shorter survival 52. Whether this is due to low T cell clonality and lack of TCR repertoire in MPM has not been investigated. T cell clonality and TCR repertoire can be used to assess the immune contexture and serve as biomarkers of T cell expansion and reactivity. In NSCLC, a heterogeneous TCR repertoire was found to be associated with poor overall survival 53 and low T cell clonality detected in lymph nodes is associated with poor prognosis in patients with cervical cancer 54. Multiregional mutational profiling of MPM tissue revealed a homogeneous genomic landscape 55. In contrast, parallel multiregional TCR sequencing of the same MPM tumors demonstrated a low T cell clonality and substantial TCR receptor heterogeneity, whereby the majority of T cell clones were restricted to individual tumor regions. Despite this, treatment of patients with the small molecule-inhibitor of SRC-family protein-tyrosine kinase dasatinib enhanced TCR clonality in MPM, which was associated with longer overall patient survival 55. Dasatinib was previously shown to improve T cell function 56. The implications of the restricted TCR repertoire in the failure to respond, at least in part, to single-agent ICB in MPM are presently not known. Moreover, how these results fit with recent data showing that combined nivolumab plus ipilimumab blockade results in improved overall survival compared to conventional chemotherapy in unresectable MPM and is approved for frontline therapy in treatment naïve patients regardless of histological subtype 57 is not yet understood.

We show that, despite overexpression of PD1 and CD127 downregulation, which binds IL-7 required for maintaining homeostatic expansion of T cells 32, MPM-derived TILs are amenable to expansion *ex vivo* in response to chronic TCR-dependent stimulation. Moreover, Ibrutinib and Rapamycin administered during periods of TCR stimulation had a pronounced effect on CD8+ and CD4+ TIL phenotype. TILs were skewed from CCR7-CD455RA- T_EM_ towards a CCR7-CD45RA+ T_EMRA_ phenotype. The re-expression of CD45RA originally has been examined mostly in CD8+ T cells and is associated with short-lived, terminally differentiated effector cells 58. Several key molecules associated with a dysfunctional phenotype also were downregulated including CTLA4, LAG3, TIGIT, DUSP4, and TOX. Interestingly, Ibr/Rap treatment significantly downregulated HLA-DR expression in both CD4+ and CD8+ TIL T_EM_ and T_EMRA_ subsets. The expression of HLA-DR on human T cells is associated with activation; however, cytotoxic effector cells were shown to be HLA-DR negative 59. Originally used to treat indolent B-cell malignancies and chronic graft versus host disease, Ibrutinib has been used to improve CAR T-cell engraftment, tumor clearance, and survival in human xenograft models of resistant acute lymphocytic leukemia and CLL when administered concurrently 42. In patients with mantle cell lymphoma in the relapsed or refractory setting, a combination of Venetoclax plus Ibrutinib shows efficacy, which was associated with expansion in CCR7-CD45RA+ CD8+ and CD4+ T_EMRA_ with improved function 60. Comprehensive immune profiling demonstrated that patients responding to combined ICB had higher frequencies of circulating CCR7-CD45RA+ CD8+ T_EMRA_ prior to the start of treatment 61.

A limitation in our study is that we examined the gene expression signatures in bulk TILs rather than separate CD8+ and CD4+ lymphocytes. Second, our study includes a small sample size, warranting confirmation in a larger cohort. Third, although CD90+ cells co-stain for well-known markers of CAFs, αSMA and FAP, and we uncovered a strong positive correlative between THY1/CD90 gene expression and CAF gene signature across TCGA MPM tumor samples estimated by multiple immune deconvolution methods (EPIC, MCPcounter, or TIDE algorithms), it is still unclear whether CD90+ cells are a mesothelial precursor cell that has undergone epithelial-to-mesenchymal transition due to protracted exposure to TGFβ1 62 or a subset of CAFs previously described in MPM 8, 63. Last, it remains to be determined whether reprogramming TILs could induce a tumor-specific immune response. Despite these limitations, our study indicates that targeting the TME together with early tuning of MPM-derived TILs achieved by targeting ITK and mTOR signaling may represent a novel approach for the treatment of MPM.

## Translational relevance

Clinical success with immune checkpoint blockade (ICB) strategies can be only observed in a small fraction of patients with malignant pleural mesothelioma (MPM), arguing that additional treatment in combination with ICB is needed. Our findings demonstrate that a high enrichment for extracellular matrix (ECM)/stromal signature in clinical MPM samples is associated with poor survival and dysfunctional tumor-infiltrating lymphocytes (TILs). Mechanistically, CD90+ cells represent a major contributor to those observations with expressing high PD-L1, enriching for ECM/stromal genes and hyperactivated PI3K-mTOR signaling with immunosuppressive features. Therapeutically, ibrutinib plus rapamycin treatment was effective in reprogramming the TIL product isolated from MPM samples to a subset of effector memory T cells that re-expresses CD45RA (termed TEMRA) with diminished expression of other exhaustion markers. Together, these findings suggest a strategy targeting the tumor microenvironment (TME) may represent a novel approach for TIL recovery leading to reactivation of anti-tumor immunity and control of tumor growth in MPM.

## Supplementary Material

Supplementary figures and tables.
