# Supplementary material for: Tumor-infiltrating lymphocytes are functionally inactivated by CD90+ stromal cells and reactivated by combined Ibrutinib and Rapamycin in human pleural mesothelioma
Source: Theranostics. 2022 Jan 1;12(1):167–85. doi: 10.7150/thno.61209 (PMC8690914; doi:10.7150/thno.61209)
Supplement: Supplementary file 1 — Supplementary figures and tables. [file thnov12p0167s1.pdf]

Supplemental Figure legends and Tables

Figure S1

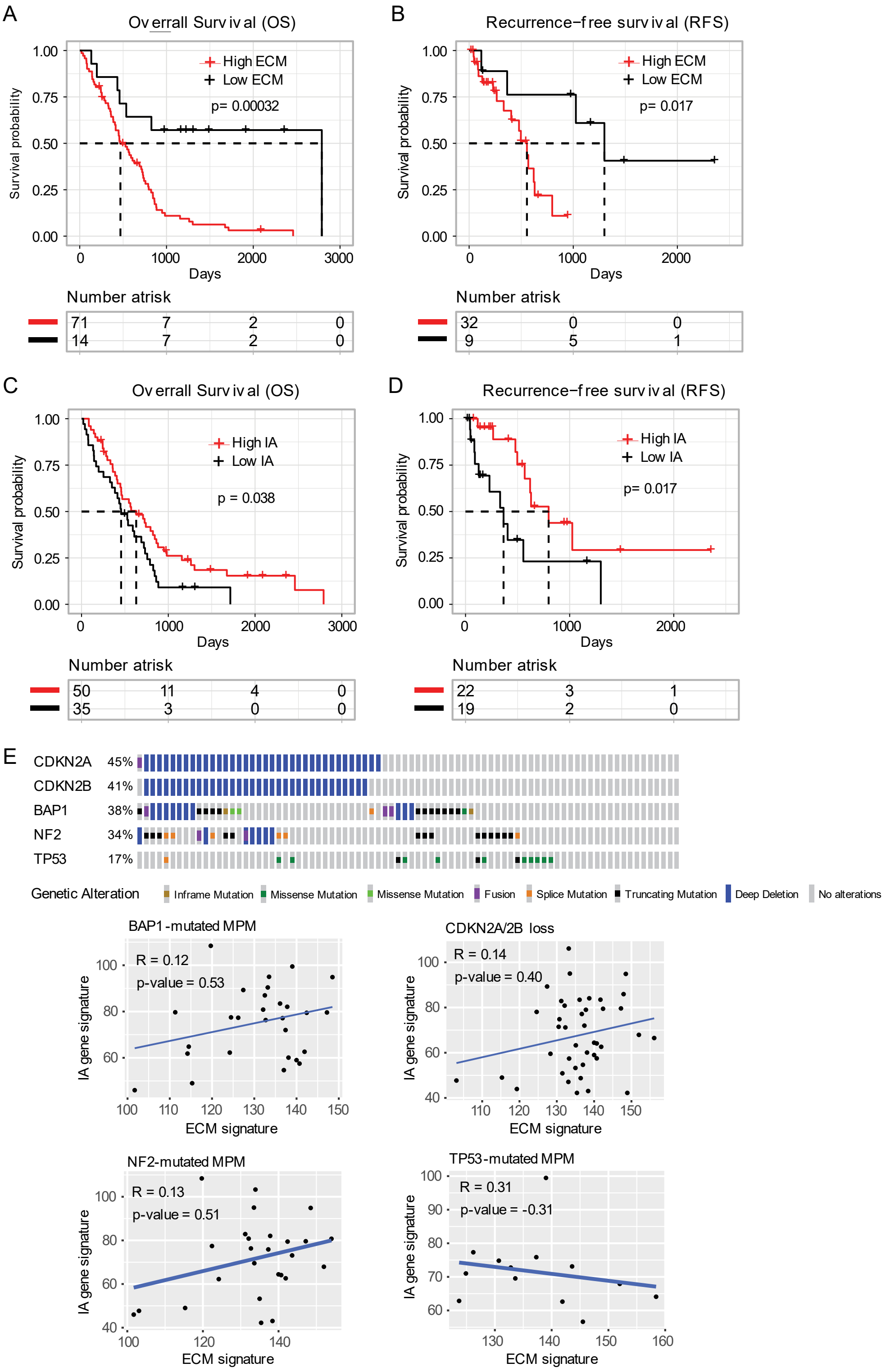

Figure S1. ECM/stromal genes and CD8 T cell activation genes are associated with prognosis in MPM. (**A-D**) Unadjusted Kaplan-Meier survival curves from TCGA

MPM cohort showing overall survival (OS) (**A, C**) and recurrence-free survival (RFS) (**B, D**) based on stratification of ECM/stromal (**A, B**) or IA gene expression (**C, D**) signature score in treatment naïve MPM. Stratification of MPM patients into high_ECM (extracellular matrix) (in red) and low ECM (in black) or high IA (in red) and low IA (in black) is based on the optimal cutoff value of ECM gene signature score and IA transcripts across all patients. (**E**) Several common mutations prevalent in MPM (upper panel) and scatter plots (lower panel) showing correlation between ECM/stromal and IA gene signatures based on several common mutations prevalent in MPM. Related to Figure 1.

Figure S2

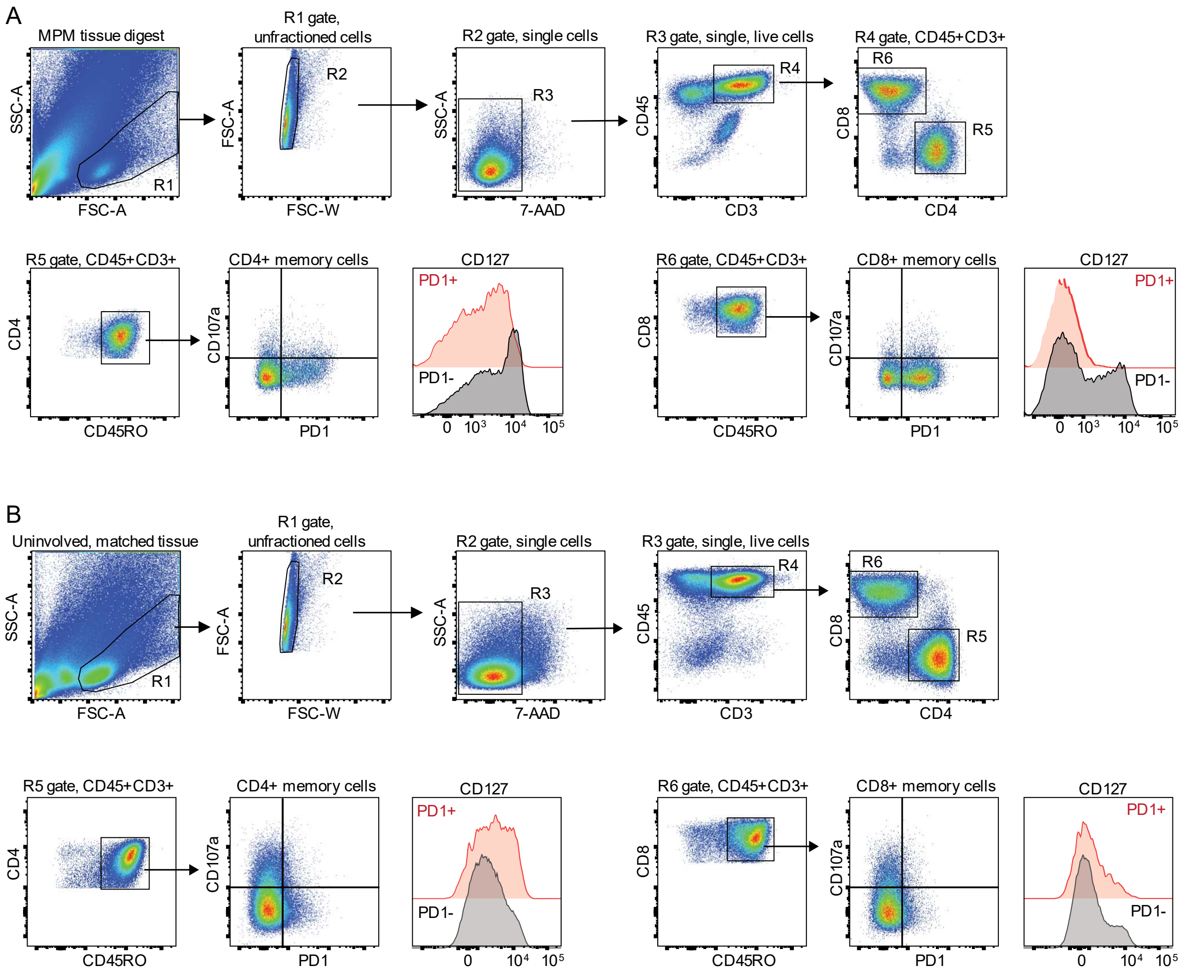

Figure S2. Flow cytometric gating strategy to identify tumor-infiltrating lymphocytes (TILs). Representative experiment showing the sequential gating strategy to identify of CD8 and CD4 T cells within the tumor (**A**) and matched (**B**) uninvolved lung tissue. R1 gate displayed on an SSC/FSC color density plot subgated to select single cells based on FSC-A versus FSC-W (gate R2), which was further subgated for identification of single live cells (7-AAD negative, gate R3). R3 gate identifying single, live cells was further subgated to show the boundary of $CD45^{+}CD3^{+}$ cells (gate R4). Single, live $CD3^{+}$ cells (gate R4) were displayed on a bivariate plot of CD4 and CD8. CD4 (gate R5) and CD8 (gate R6) cells were then each separately displayed as a bivariate plot to identify CD45RO+ memory T cells and their coexpression of PD1 and CD107a and CD127. Related to Figure 2.

Figure S3

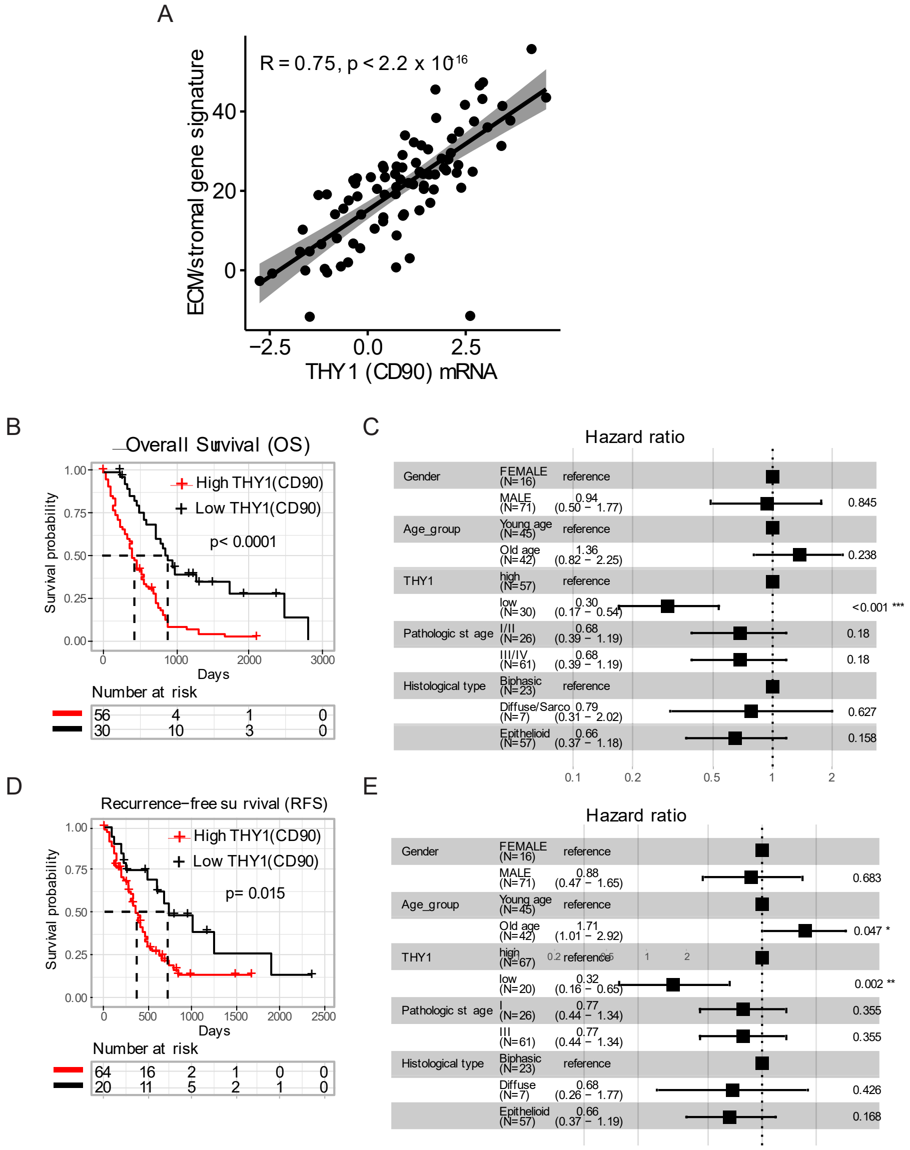

Figure S3. CD90 expression is an independent factor predicting survival of MPM patients.

(**A**) Correlation analysis of CD90 mRNA level and ECM/stromal gene signature across The Cancer Genome Atlas (TCGA) MPM cohort. (**B-E**) Univariate (**B, D**) and multivariate analyses (**C, E**) showing overall survival (OS) (**B, C**) and recurrence-free survival (RFS) (**D, E**) stratified by the gene expression of CD90 in treatment naïve MPM. Related to Figure 4.

Figure S4

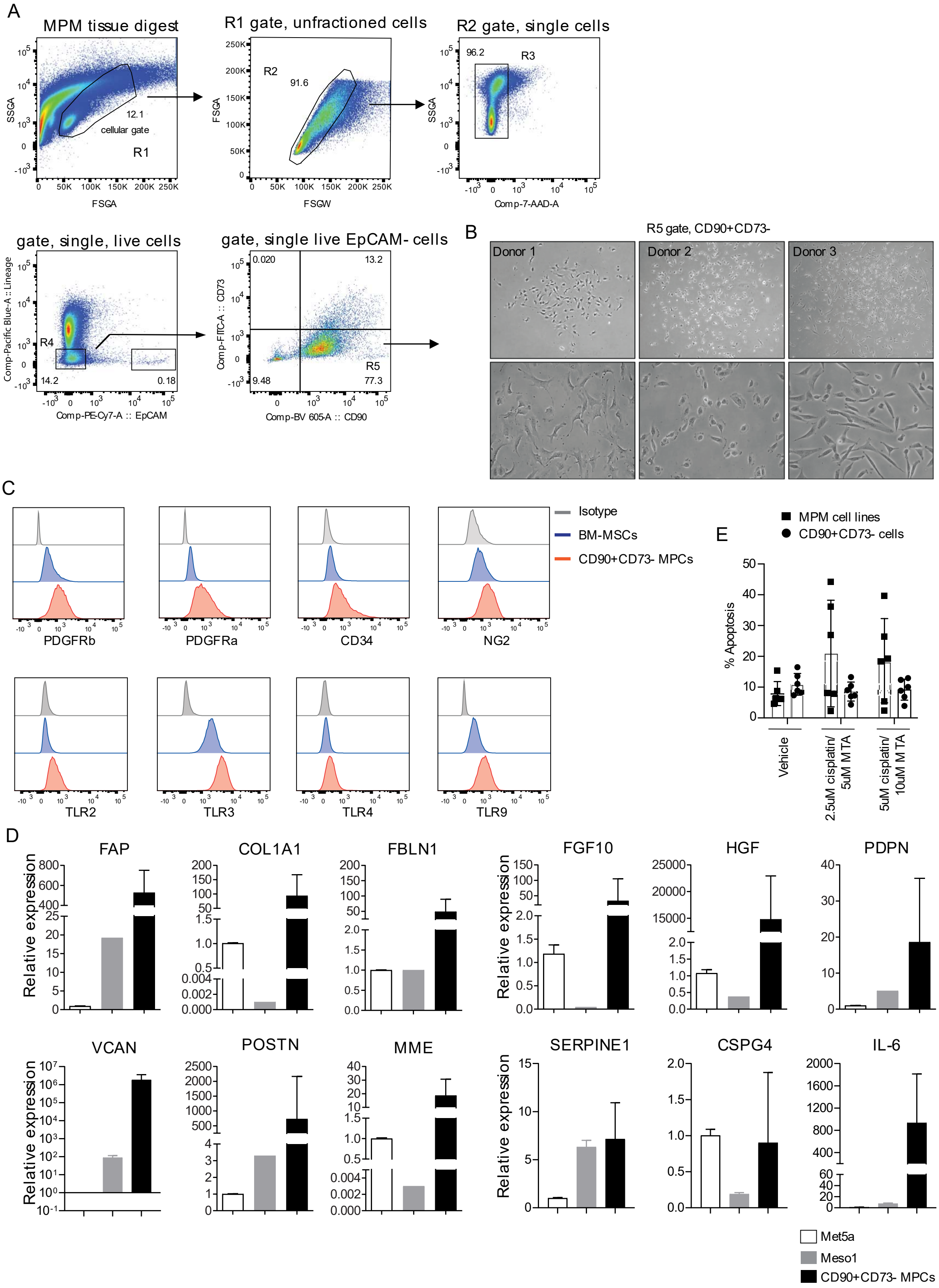
A
MPM tissue digest
R1 gate, unfractioned cells
R2 gate, single cells
cellular gate
R1
R2
R3
R4
R5
gate, single, live cells
gate, single live EpCAM- cells
B
R5 gate, CD90+CD73-
Donor 1
Donor 2
Donor 3
C
Isotype
BM-MSCs
CD90+CD73- MPCs
PDGFRb
PDGFRa
CD34
NG2
TLR2
TLR3
TLR4
TLR9
E
MPM cell lines
CD90+CD73- cells
% Apoptosis
Vehicle
2.5uM cisplatin/ 5uM MTA
5uM cisplatin/ 10uM MTA
D
FAP
COL1A1
FBLN1
FGF10
HGF
PDPN
VCAN
POSTN
MME
SERPINE1
CSPG4
IL-6
Relative expression
Met5a
Meso1
CD90+CD73- MPCs

Figure S4. **Identifying and characterizing CD90+ cell subset in MPM.** (**A**) Gating strategy to identify mesenchymal cell fractions in MPM and their subsets expressing CD90 and CD73 shown as bivariate plots from one representative donor. (**B**) Phase contrast images of prospectively isolated CD90+ cells using FACS. (**C**) Representative histograms showing expression of several surface proteins in CD90+ MPCs. (**D**) Box plots showing the change in mRNA expression in a distinct ECM/stromal gene set in CD90+ MPCs. mRNA level in MET-5A mesothelial cells is set at one. CD90+ MPCs, n = 10, biological replicates in total. (**E**) Bar graph showing the quantification of cells stained for Annexin V and propidium iodide (PI) after exposure to chemotherapy. MPM cell lines, n = 6, (Meso1, Meso44, H28, JL1, H2452, H2052) and CD90+CD73- cells, n = 6, biological replicates. Data are presented as mean ± SD. Error bars show SD. Analysis of more than two groups by one-way ANOVA and multiple comparisons using post hoc Tukeys test. Related to Figure 4.

Figure S5

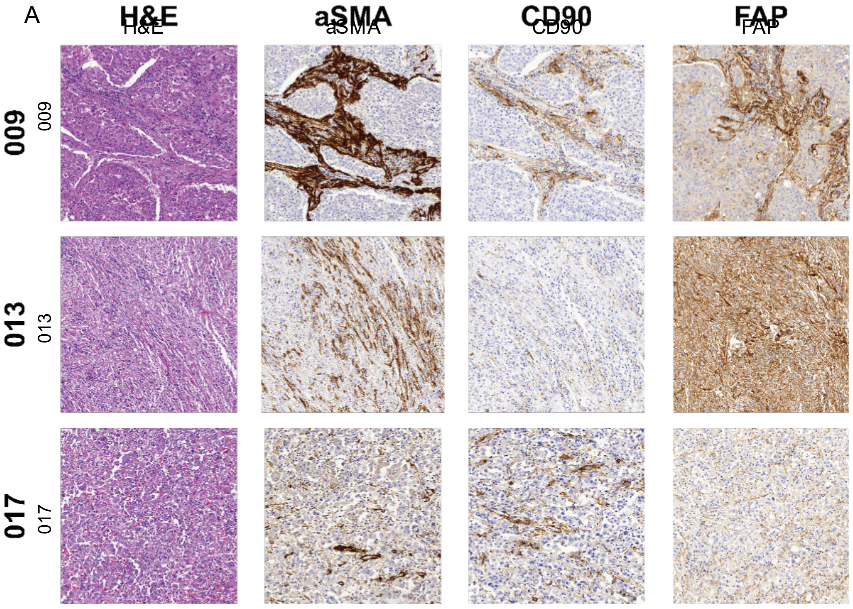
A
H&E
aSMA
CD90
FAP
H&E
aSMA
CD90
FAP
009
009
013
013
017
017

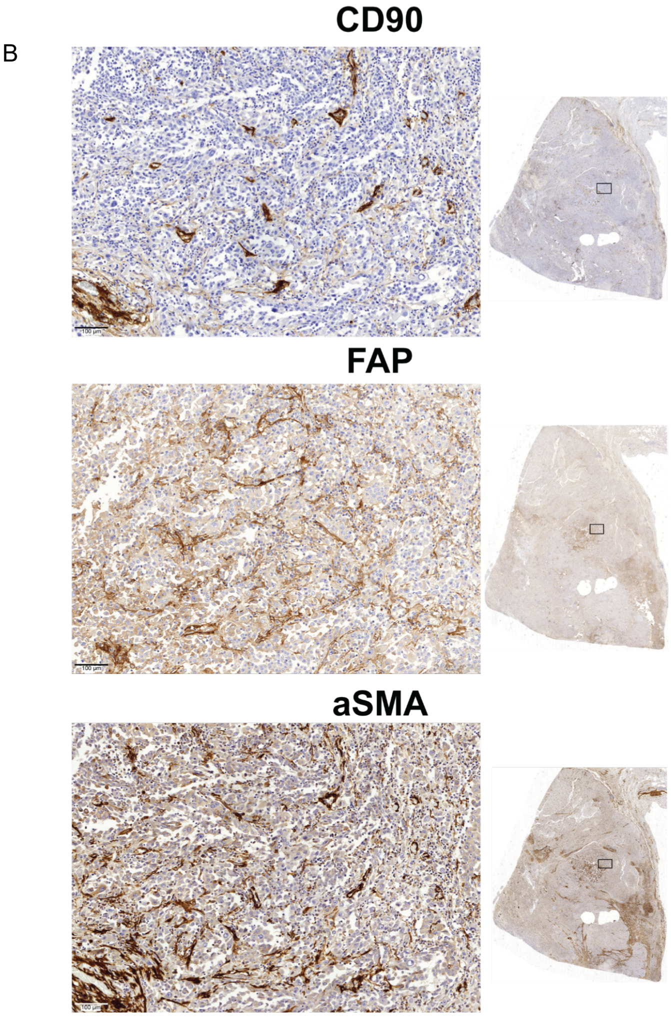
B
CD90
FAP
aSMA

Figure S5. CD90+ cells co-express αSMA and FAP. (**A**) Post-NAC samples in hematoxylin & eosin (H&E), alpha-smooth muscle actin (αSMA), CD90 and fibroblast activation protein (FAP). (**B**) Co-expression in CD90, αSMA and FAP in the post-NAC sample of an epitheloid specimen 017. Scale bar, 100 μm. Related to Figure 4.

Figure S6

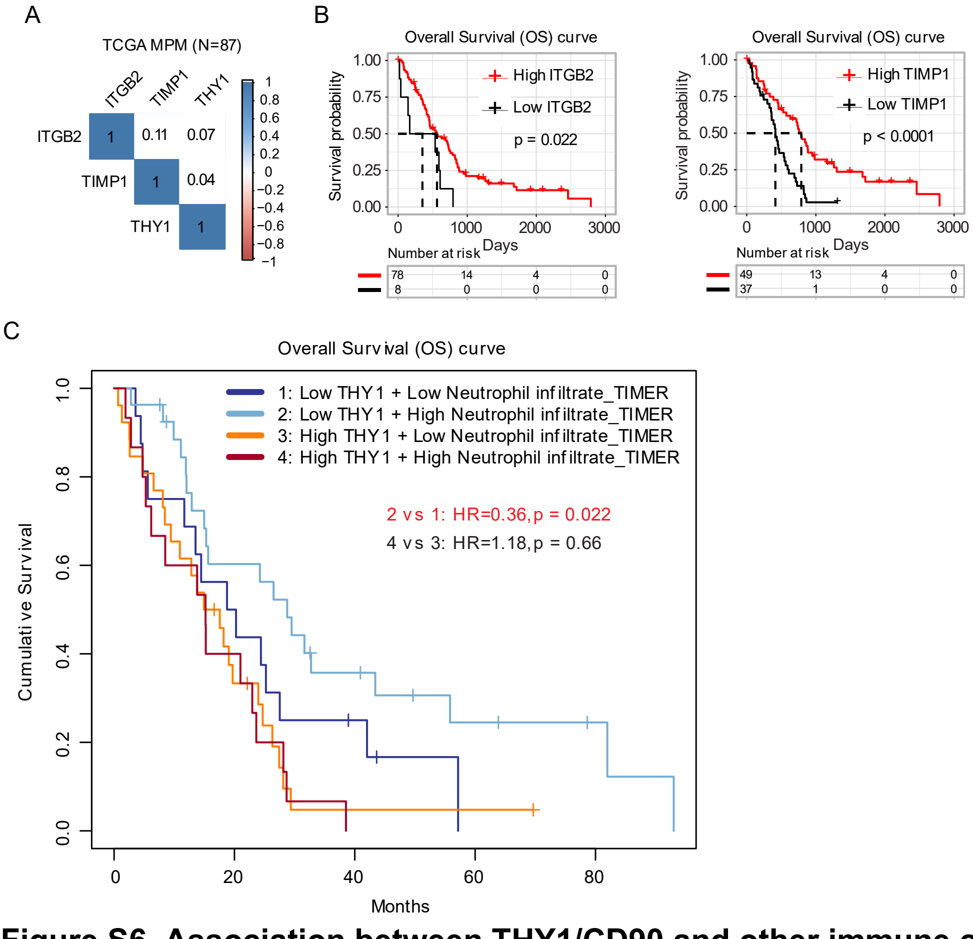

Figure S6. Association between THY1/CD90 and other immune cell markers. (**A**) Correlation matrix showing correlation among THY1, ITGB2 (Mac-1), and TIMP1 expression. (**B**) Kaplan–Meier analysis of TCGA MPM patient cohort based on ITGB2 and TIMP1 expression. Patients with high- (red) or low (black) gene expression were stratified by the optimal cutoff value of the ITGB2 and TIMP1, respectively, across all patients using the surv_cutpoint function in the R "maxstat" package. Overall survival

rates were analyzed and plotted using the R "survival" and "survminer" package. The p-value is calculated by the log-rank test. (**C**) Cox multivariate analysis showing the association of THY1 expression and neutrophil infiltrates with overall survival in TCGA MPM patients. Age, gender, tumor stage and tumor purity were adjusted. Related to Figure 4.

Figure S7

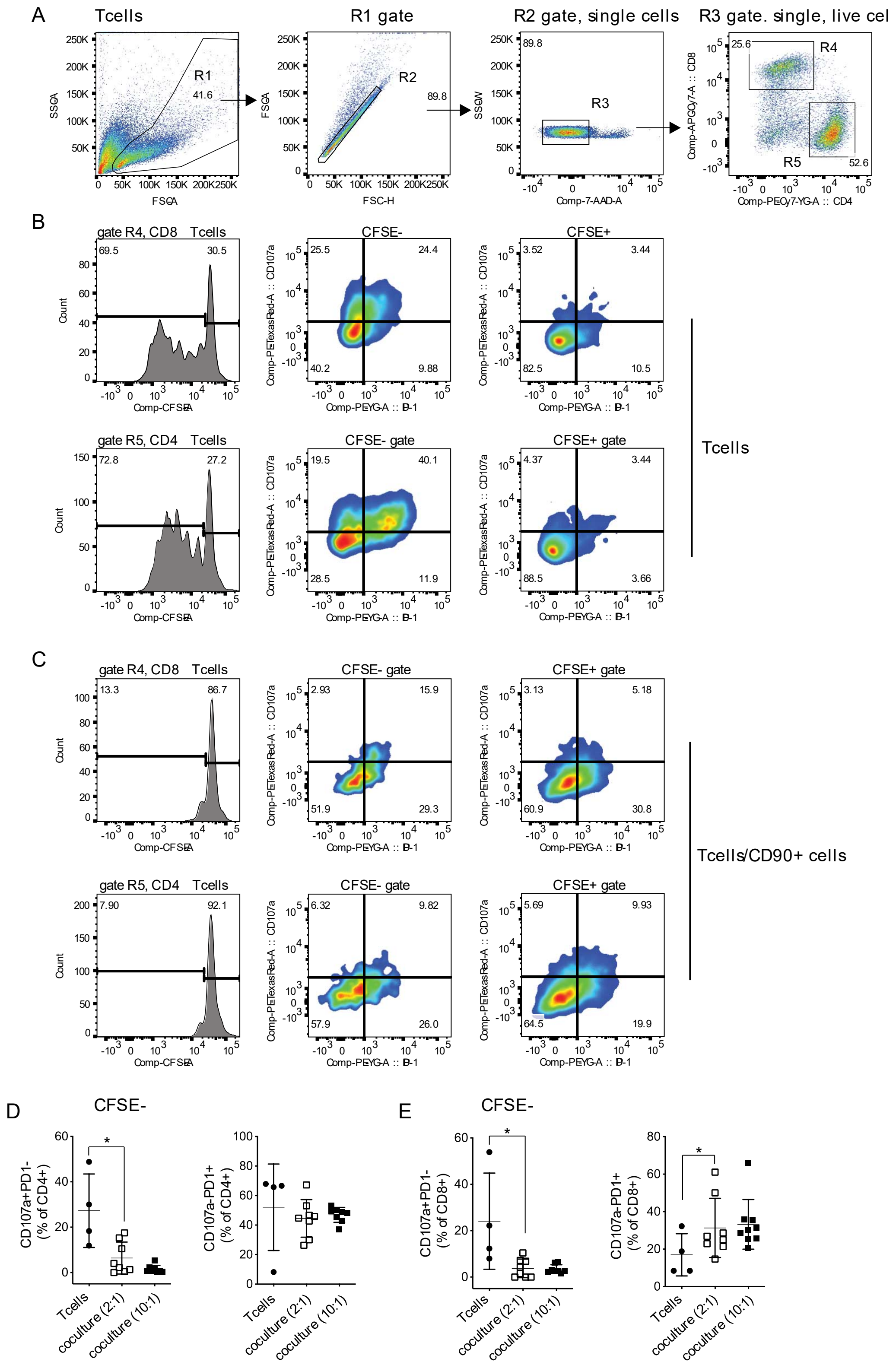

Figure S7. CD90+ MPCs inhibit T cells following TCR-dependent stimulation with SEB. (**A**) Schematic representation of gating strategy to examine the immune

suppressive function of CD90+ MPCs cells on CD3+ T cells. (**B, C**) Representative flow cytometric histograms showing proliferation of CFSE-labeled T cells and expression CD107a and PD1 isolated from the peripheral blood of a healthy donor 5 days following activation with staphylococcal enterotoxin B (SEB, 500 ng/mL) alone (**B**) or in the presence of immune primed (IP) CD90+ MPCs cells (**C**) at a 2:1 ratio. (**D, E**) Scatter plots showing % of proliferating (CFSE-) CD4 and CD8 T cells (**D**) and non-proliferating (CFSE+) CD4 and CD8 T cells (**E**). For coculture conditions n = 9 in total. Data presented as mean ± SD. Significant differences calculated using one way ANOVA following by post hoc Tukey's range test. * $P < 0.05$; ns, not significant. Related to Figure 5.

Table S1. Patient Clinical Characteristics

**Total # of patients, n**	20
**Gender, n (%)**	
male	17 (85%)
female	3 (15%)
**Age at diagnosis, years, mean (range)**	71 (64 – 81)
**Histology**	
Epitheloid	14 (70%)
Biphasic	3 (15%)
Sarcomatoid	2 (10%)
Epitheloid/Sarcomatoid	1 (5%)
**Asbestos exposure**	
yes	8 (40%)
no	10 (50%)
unknown	2 (10%)
**Smoking Status, n (%)**	
never	8 (11%)
former	10 (85%)
current	4 (67%)
unknown	2 (3%)
pack years, average (range)	20 (1-60)
**Prior chemotherapy, n (%)**	
yes	16 (80%)
no	4 (20%)
**Patient survival, n (%)**	3 (15%)

Table S2. List of antibodies used for FACS/flow cytometric analysis

Primary Antibodies for Flow Cytometry	Manufacturer	Clone	Catalog #
CD326-PE-Cy7	eBioscience	1B7	25-9326-42
CD73-APC	eBioscience	AD2	17-0739-42
CD73-APC-Cy7	BioLegend	AD2	344022
CD90-BV605	BioLegend	5E10	328128
CD90-PE-TR	BD Biosciences	5E10	562385
CD45-eFluor®450	eBioscience	2D1	48-9459-42
CD14-eFluor®450	eBioscience	61D3	48-0149-42
CD31-eFluor®450	eBioscience	WM-59	48-0319-42
CD235a-eFluor®450	eBioscience	HIR2	48-9987-42
PD-L1-APC	eBioscience	M1H1	17-5983-42
PD-L1-PE	eBioscience	M1H1	12-5983-42
CD45-Percp-Cy5.5	eBioscience	2D1	45-9459-42
CD19-FITC	BioLegend	HIB19	302206
CD3-eVolve™605	eBioscience	OKT3	83-0037-42
CD3-APC-R700	BD Biosciences	UCHT1	565119
CD3-BV421™	BioLegend	OKT3	317344
CD4-PE-Cy7	eBioscience	RPA-T4	25-0049-42
CD4-BV421	BioLegend	RPA-T4	300531
CD4-BUV396	BD Horizon™	RPA-T4	564724
CD8-APC/Fire™750	BioLegend	SK1	344746
CD8-APC-eFluor®780	eBioscience	SK1	47-0087-42
CD8-BUV	BD Horizon™	RPA-T8	612942
CD45RO-APC	eBioscience	UCHL1	17-0457-42
CD45RA-PE-Cy7	BioLegend	HI100	304126

CD45RA-APC	eBioscience	HI100	17-0458-42
CCR7-BV421™	BioLegend	G043H7	353208
PD-1-PE	eBioscience	eBioJ105	12-2799-42
CD107a-PE-eFluor™610	eBioscience	EBioH4A3	61-1079-42
CD107a-PE	BioLegend	H4A3	328608
CD127-BV711	eBioscience	EBioRDR5	61-1278-42
CD127-eFluor®450	eBioscience	EBioRDR5	48-1278-42
HLA-DR-FITC	eBioscience	LN3	11-9956-42
TOX-PE	eBioscience	TXRX10	12-6502-82
CTLA4-BV786	BD Horizon™	BNI3	563931
NG2-Alexa Fluor 488	eBioscience	9.2.27	53-6504-82
CD34-PE	Miltenyi Biotec	AC136	130-113-179
PDGFRα-PE-Cy7	BioLegend	16A1	323508
PDGFRβ-APC	BioLegend	18A2	323608
TLR2 (CD-222)-BV421	BD Horizon™	11G7	565350
TLR3 (CD283)-PE	eBioscience	TLR3.7	12-9039-82
TLR4 (CD284)-BV786	BD Horizon	TF901	564402
TLR9 (CD286)-APC	eBioscience	eB72-1665	17-9099-82
7-AAD	eBioscience		00-6990-50
Live/Dead Near IR	eBioscience		L34975
Zombie green	BioLegend		432112

Table S3. List of primer probes

**Target gene**	**Refseq ID**
FGF10	NM_004465(1)
PDPN	NM_001006624(2)
HGF	NM_000601(2)
POSTN	*NM_001135934.1*
IL6	*NM_000600.3*
FAP	NM_001291807.1
VCAN	NM_001126336.2
COL1A1	NM_000088.3
FBLN1	NM_001996.3
MME	NM_000902.3
SERPINE1	NM_000602.4
CSPG4	NM_001897.4
**Internal control**	**Refseq ID**
HMBS	NM_000190(1)
TBP	*NM_003194*
POLR2A	*NM_000937*
PPIA	NM_021130(1)
ACTB	NM_001101(1)
HMBS	NM_000190(1)

Table S4. Co-expression of CD90 with fibroblast markers alpha smooth muscle actin (αSMA) and fibroblast activation protein (FAP) in the different tiles of samples.

ID	CD90/aSMA	CD90/FAP
009_post_1	complete	complete
009_post_2	complete	complete
009_post_3	complete	complete
009_pre_1	complete	complete
009_pre_2	complete	complete
009_pre_3	complete	complete
013_post_1	complete	complete
013_post_2	complete	complete
013_post_3	complete	complete
013_pre_1	complete	complete
013_pre_2	partial	
013_pre_3	complete	complete
017_post_1	complete	complete
017_post_2	complete	complete
017_post_3	complete	complete
017_pre_1	complete	
017_pre_2	partial	complete
017_pre_3	complete	complete

Complete, complete co-expression; partial, partial co-expression; non, no co-expression
